# Exosomes derived from cancer-associated fibroblasts promote tumorigenesis, metastasis and chemoresistance of colorectal cancer by upregulating circ_0067557 to target Lin28

**DOI:** 10.1186/s12885-023-11791-5

**Published:** 2024-01-12

**Authors:** Cheng Yang, Yan Zhang, Mingze Yan, Jiahao Wang, Jiaming Wang, Muhong Wang, Yuhong Xuan, Haiyue Cheng, Jiaao Ma, Cuicui Chai, Mingzhe Li, Zhiwei Yu

**Affiliations:** 1 Department of Colorectal Surgery, Harbin Medical University Cancer Hospital, 150086 Harbin, China; 2 Digestive Disease Center, The Seventh Affiliated Hospital of Sun Yat-sen University, 518107 Shenzhen, China

**Keywords:** Cancer-associated fibroblasts, Exosomes, Colorectal cancer, circ_0067557, Lin28A, Lin28B

## Abstract

**Background:**

Cancer associated fibroblasts (CAFs) can remodel tumor microenvironment by secreting exosomes. This study aimed to investigate the role of exosomes derived from cancer-associated fibroblasts in colorectal cancer (CRC) progression.

**Methods:**

Circular RNA (circRNA) array was used to identify differentially expressed circRNAs in exosomes from normal fibroblasts (NFs) and CAFs, and confirmed one differentially expressed circRNA circ_0067557 by real-time PCR. The effect of circ_0067557 on proliferation, metastasis, chemoresistance and apoptosis was verified by wound heal, tranwell, CCK8, sphere-forming and flow cytometry assay.

**Results:**

Circ_0067557 expression in exosomes from CAFs was higher than those from NFs. CAF-derived exosomes promoted the proliferation, migration, invasion and chemoresistance of CRC cells while suppressed apoptosis. Silencing of circ_0067557 inhibited malignant phenotypes of CRC cells by targeting Lin28A and Lin28B. Moreover, CAF-derived exosomes enhanced the growth of CRC xenograft tumors.

**Conclusion:**

Circ_0067557/Lin28A and Lin28B signal axis may be a potential therapy target for CRC.

**Supplementary Information:**

The online version contains supplementary material available at 10.1186/s12885-023-11791-5.

## Background

Colorectal cancer (CRC) is a common tumor of the digestive system worldwide [1]. Statistically, there were 1.93 million new cases of CRC in the global population in 2020, ranking the third in malignancies and the second in cancer-related deaths [2]. The high mortality of CRC is due to tumor metastasis and chemoresistance which lead to the main therapy failure in CRC [3, 4]. CRC development is a complex multi-step process involving RNA regulation, DNA damage repair, epigenetic changes, and mutations in oncogenes and tumor suppressors [5, 6]. In the past decade, great attention has been focused on the role of the tumor microenvironment in tumor progression and chemoresistance [7].

Cancer associated fibroblasts (CAFs) are abundant stromal cells in tumor microenvironment (TME) and remodel the extracellular matrix and TME by secreting exosomes [8, 9]. CAFs and tumor cells can exchange information through exosomes to regulate tumor progression [10, 11]. For instance, CAFs can affect adhesion, endocytosis, and the crosstalk with esophageal carcinoma cells by secreting exosomes [12]. CAF-derived exosomes can also modulate proliferation and gemcitabine resistance in pancreatic cancer [13]. However, the role of CAF-derived exosomes in malignant progression and chemoresistance of CRC is still elusive.

Exosomes participate in cell-to-cell interactions by transmitting intracellular cargos, including functional proteins, messenger RNAs (mRNAs), microRNAs (miRNAs), Circular RNAs (circRNAs) and long non-coding RNAs (lncRNAs) [14, 15]. Recent studies found that circRNAs are enriched and exist stably in exosomes, and may be used as biomarkers for diagnosis of human diseases [16, 17]. Circular RNAs (circRNAs) are special RNAs characterized by a covalently closed loop and highly stable compared with parental linear RNAs [18], circRNAs can alter tumor proliferation, metastasis, and drug resistance, providing novel markers and targets for early diagnosis and treatment [19, 20]. Therefore, in this study we aimed to investigate the role of circRNAs in exosomes derived from CAFs in CRC progression and chemoresistance.

Our results demonstrated the impact of CAF-derived exosomes on tumorigenesis, metastasis and chemoresistance of CRC cells. We identified differentially expressed circRNAs (DECs) in exosomes from CAFs using circRNA microarray, and elucidated the mechanism by which circ_0067557 regulated CRC progression.

## Methods

### Patients and samples

CRC and adjacent normal tissues were archived from 12 patients with CRC who had undergone surgical resection at Harbin Medical University Cancer Hospital from March 2019 to April 2020. Among the 12 patients, 6 were male and 6 were female. Their mean age was 62 years, including 7 over 60 years old and 5 under 60 years old. The tumor stage was I in 4 patients, II in 4 patients and III in 4 patients. Six patients had tumor located in the colon and 6 patients had tumor located in the rectum. Each CRC patient had the disease confirmed by pathology when first diagnosed. The patients had no other malignancies or autoimmune diseases and provided written informed consent. This study was approved by the Ethics Committee of Seventh Affiliated Hospital of Sun Yat-Sen Zhongshan University and followed Declaration of Helsinki.

### Cell culture

HCT116 (CCL-247), Lovo (CCL-229), and SW480 (CCL-228) cells were purchased from ATCC, and grown in DMEM (Gibco, USA), F12K (Invitrogen, USA), and L-15 (Gibco) medium, respectively, supplemented with 10% fetal bovine serum (FBS, Sigma, Germany). All cells were cultured in a 37°C; incubator with 5% CO_2_.

### Cell transfection and treatment

Empty vector (pcDNA 3.1), circ_0067557-, and the Lin28A- and Lin28B-overexpressed plasmids were obtained from HanBio Biotechnology (HanBio, Shanghai, China). Circ_0067557 siRNAs (sicirc_0067557) and negative control (siNC) were acquired from Genepharma (Shanghai, China). The sequences for sicirc_0067557#1 were 5’-GUGGUCUGCAAGCAAGUAUUU-3’’ and 5’-UUCACCAGACGUUCGUUCAUA-3’’. The sequences for sicirc_0067557#2 were 5’’- CAAGGAAUCAGCAGGAUGUUU-3’ and CAAGGAAUCAGCAGGAUGUUU-3’and 5’-UUGUUCCUUAGUCGUCCUACA-3’’ The sequences for sicirc_0067557#3 were 5’-GUCUGUCUCUUGGCUUUGUUU-3’ and 5’-GUCUGUCUCUUGGCUUUGUUU-3’ and 5’’- UUCAGACAGAGAACCGAAACA-3’’. HCT116, Lovo, and SW480 cells (5 × 10^5^ cells/mL) were plated into 6-well plates. At 80% confluence, the cells were transfected with the plasmids using Lipofectamine 3000 (Invitrogen) and cultured for 48 h.

### Chemoresistance assay

The cells were treated with 2 µM 5-Fluorouracil (5-FU) and 2 µM Oxaliplatin (L-OHP) based on previous study [21]. The cells were collected for CCK8 assay at different time points (24, 48 and 72 h).

### Isolation of normal fibroblasts (NFs) and CAFs

The fresh CRC tumors and adjacent normal tissue were washed three times with sterile PBS, cut into pieces, and digested with a mixed solution of collagenase (Cat. No. SCR103; MERCK/Sigma-Aldrich, Darmstadt, Germany), neutral protease (Cat. No. C0773; MERCK/Sigma-Aldrich, Darmstadt, Germany), and hyaluronidase (Cat. No. H1115000; MERCK/Sigma-Aldrich, Darmstadt, Germany) for 40 min. After centrifugation at 500 rpm for 5 min, the samples were resuspended in 12% FBS medium and seeded in cell culture dishes. After three days, the medium was replaced to remove tissue fragments and non-adherent cell. The epithelial cells were removed by differential adhesion methods, leaving fibroblasts.

### Immunofluorescence (IF) staining

CAFs were fixed with 4% paraformaldehyde (PFA, Cat. No.158,127; MERCK/Sigma-Aldrich, Darmstadt, Germany) for 30 min and then permeabilized with Triton X-100 (0.01%, Thermo Fisher Scientific, MA, USA) for 10 min. After blocking with 0.1% bovine serum albumin (BSA), the cells were incubated with primary antibody overnight at 4°C. The primary antibodies were as follows: FAP (1:100, #66,562, Cell Signaling Technology/CST, MA, USA), Vimentin (1:150, #5741, CST, MA, USA), and α-SMA (1:100, #19,245, CST, MA, USA). After washing, the samples were incubated with goat anti-rabbit IgG (Alexa Fluor® 488, ab150077, Cambridge, UK) for 30 min. The cells were counterstained with DAPI (1 mg/mL, Sigma-Aldrich, Missouri, USA) and blocked with glycerin. Staining was visualized using a fluorescence microscope (Lionheart LX, BioTeK, Vermont, USA).

### Exosome extraction

To collect the exosomes secreted by CAFs, the medium was collected and filtered with a 0.22 μm filter (SLGP033N, Merck-Millipore, NJ, USA) as previously described [22]. The NF- and CAF-derived exosomes were isolated using ExoQuick-TC Exosome Precipitation (EXOTC50A-1, System Biosciences, Palo Alto, CA).

### Transmission electron microscopy (TEM)

The extracted exosomes were suspended in 0.2% PFA (Sigma-Aldrich), and the suspension (10 µL) was dropped onto a hydrophilic copper mesh for 5 min. The samples were stained using 1.75% uranyl acetate for 2 min. After natural drying, the exosomes were observed using TEM (HITACHI H-7 500, Hitachi, Tokyo, Japan).

### Dynamic light scattering (DLS)

The size of the exosomes was determined using Nanosizer™ technology (Malvern Instruments, Malvern, UK) and Zetasizer software (Malvern Panalytical, Malvern, UK) [23].

### Real-time PCR (RT-qPCR)

Total RNA was isolated from CRC tissues and cells using TRIzol reagent (Invitrogen, MA, USA). The RNA was reverse-transcribed into cDNA using PrimeScripTM (Takara, Tokyo, Japan). PCR amplification was performed using SYBR Green qPCR Master Mix (DBI Bioscience, Germany). The relative gene expression levels were calculated using the 2^−△△CT^ method. All primer sequences are presented in Supplemental Table 1. GAPDH was used as an internal reference.

### Arraystar circRNA microarray

CircRNA microarray analysis was conducted by Kangcheng Bio-Tech (Hangzhou, China). The RNAs isolated from 12 samples of CRC tissues were treated with Rnase R to remove linear RNAs and enrich circRNAs. The samples were then analyzed using an Agilent Bioanalyzer. The circRNAs were amplified using random primers, labeled, and purified with a RNeasy Mini Kit. The concentration of the labeled circRNAs was measured with a Nanodrop 2000. The labeled circRNAs (1 µL) were incubated with 20 µL hybridization buffer (2×) to stop the fragmentation reaction. The hybridization solution (50 µL) was dispersed onto the circRNA microarray, which was then hybridized at 65°C for 17 h. After hybridization, the chips were washed, fixed, and scanned using an Agilent Scanner (G2505C, OE Biotech. Co., Ltd., Shanghai, China).

### Bioinformatics analysis

EnhancedVolcano package in R software was used to draw PCA-VOLcano plots, and ComplexHeatmap package in R software was used to draw heatmap. TopGO version 2.32.0 was used for Gene Ontology (GO) enrichment analysis of DECs. Kyoto Encyclopedia of Genes and Genomes (KEGG) enrichment analysis of DECs was performed based on KEGG website (https://www.genome.jp/kegg/).

### Western blotting

Total protein was isolated using RIPA buffer (P0013E, Beyotime, Shanghai, China), separated by electrophoresis, and transferred onto PVDF membranes (Millipore). The blots were incubated overnight at 4°C with primary antibodies for Lin28A (1: 1000; Abcam, ab279647), Lin28B (1: 2000; Abcam, ab191881), CD63 (1: 1000; Abcam, ab271286), ALIX (1: 1000; Abcam, ab275377), Calhexin-CNX (1: 1000; Abcam, ab133615), CD44 (1: 1000; Abcam, ab189524), CD133 (1: 2000; Abcam, ab222782), OCT4 (1: 1000; Abcam, ab19857), ALCAM (1: 1000; Abcam, ab279580), Vimentin (1: 1000; Abcam, ab8978), E-Cadherin (1: 1000; Abcam, ab231303), CyclinD1 (1: 200; Abcam, ab16663), C-Myc (1: 1000; Abcam, ab32072), and GAPDH (1: 10,000; Abcam, ab8245). After washing, the blots were incubated with secondary antibody HRP Goat anti-Rabbit IgG (1: 1000; BA1054, BOSTER, Wuhan, China) or HRP Goat anti-Mouse IgG (1: 1000; BA1051, BOSTER, Wuhan, China) for 1 h, and the staining was visualized by enhanced chemiluminescence (ECL, Millipore). The blots were cut prior to hybridization with antibodies during blotting and original blots with membrane edges visible were shown in Supplementary files.

### CCK-8 assay

CCK-8 assay was performed following the protocols of CCK-8 kit (Dojindo, Tokyo, Japan). CRC cells (1 × 10^4^ cells/well, 100 µL) were grown in 96-well plates and then subjected to CCK-8 assay at 24, 48, and 72 h. At the designated time point, 20 µL CCK-8 solution was added to each well, and the cells were cultured for 3 h. After the incubation, the absorbance of the wells at 450 nm was measured using a microplate reader (Bio-Tek Epoch, Bio-Tek, VT, USA).

### 5-Ethynyl-2^,^-deoxyuridine (EdU) assay

Edu assay was performed following the protocols described previously [24]. Briefly, cells were incubated with EdU (10 µmol/L) for 2 h in serum-free medium. The cells were stained with Hoechst 33,342 (H-33,342), and imaged by fluorescence microscopy (OLYMPUS, Japan).

### Sphere-forming assay

Sphere-forming assay was performed as described previously [25]. CRC cells were seeded into 12-well plates and cultured for seven days at 37°C. After washing with PBS, the cells were fixed for 15 min with 4% PFA and stained with 0.2% crystal violet for 10 min. After drying, the colonies with diameter > 50 μm were counted after photographing with a light microscope (OLYMPUS, CX41, USA).

### Flow cytometry

CRC cells were harvested, centrifuged, and resuspended in 1× binding buffer (100 µL). The cells (1 × 10^6^) were stained with 5 µL FITC-Annexin V and 5 µL PI for 15 min in the dark. The rate of apoptosis was measured using the FACSCalibur Flow cytometer (BD Biosciences, NJ, USA).

### Wound healing assay

Wound healing assay was performed as described previously [26]. CRC cells (approximately 1 × 10^6^/well) were seeded into 24-well plates and cultured at 37°C for adhesion. A wound was created by scratching with vertical pipette in the monolayer of cells. After culture in serum-free medium for 48 h, images of cells migrating into the wound area were captured. The relative wound area was calculated at 0 and 48 h.

### Transwell assay

Transwell assay was performed as described previously [27]. The upper chambers of the Transwells (Costar; MA, USA) were coated with Matrigel (50 mg/L, Cat. No. 356,234, CORNING, NYS, China). 100 µL of CRC cells suspension (2 × 10^5^/mL) were seeded into the upper chamber, and complete medium (600 µL) was added to the lower chambers. After 24 h, the cells were fixed and stained with 0.2% crystal violet (Sigma; C0775) for 30 min. The number of invading cells were counted in five random microscopic field, and the average value calculated.

### RNA immunoprecipitation (RIP) assay

The RIP assay was conducted using the Protein Immunoprecipitation Kit (Millipore, USA). Briefly, HCT116 cells were lysed using RIP lysis buffer containing magnetic beads conjugated with anti-Lin28A or anti-Lin28B antibody (produced by Miltenyi Biotech, German) or normal IgG (Millipore, USA). The levels of the co-precipitated RNA (i.e., circ_0067557) were detected by RT-qPCR.

### RNA-pull down assay

To confirm a possible interaction between circ_0067557 and Lin28A or Lin28B, treated streptavidin-conjugated magnetic beads (100 µL) were incubated with 0.5 g/L yeast tRNA and 1% RNAse-free BSA and then biotinylated circ_0067557 (100 pmol) or control probes (25 µL) at 4°C for 3 h. The biotinylated circ_0067557-bound beads were incubated with 750 µL lysate at 4°C for 3 h and then centrifuged. An aliquot of the supernatant (50 µL) was removed as input. After binding, the RNA complex was eluted and Lin28A and Lin28B were detected by Western blotting.

### Electrophoretic mobility shift assay (EMSA) assay

EMSA probes were designed and synthesized by Boxin Biotechnology Co. (Guangzhou, China). Nuclear proteins were harvested using a nuclear protein extraction kit (Cat. No.71,282, Sigma-Aldrich) according to the manufacturer’s instructions. The nuclear protein concentrations were determined using the Pierce™ BCA kit (Thermo Fisher Scientific), and nuclear proteins were electrophoresed on a 6.5% SDS-PAGE gel at 200 V for 40 min and then visualized under UV light after being stained with SYBR green (Molecular Probe).

### Tumor xenograft model

BALB/c nude mice (SPF, male, 4-weeks, 20 ± 2 g) were obtained from Shanghai Slack Laboratory Animal Co., Ltd. Mice were housed at 22–25°C, 45–55% humidity with a 12 h light cycle and freely available food and distilled water. The animal experiments were approved by the Ethics Committee of Seventh Affiliated Hospital of Sun Yat-Sen Zhongshan University. HCT116 cells (5 × 10^6^ cells in PBS) were injected subcutaneously into the axillary region of nude mice. After the tumor grew to 50–100 mm^3^, nude mice were randomly divided into five groups (*n* = 6): HCT116, HCT116 + CAFs exo, HCT116 + 5-FU / L-OHP, HCT116 + CAFs exo + 5-FU / L-OHP, and HCT116 + shcirc_ 0067557 + CAFs exo + 5-FU / L-OHP. Mice in HCT116 group were injected with 100 µL normal saline, and mice in other groups were injected with CAFs exo (10 µg, 100 µL) and/or 5-FU / L-OHP (10 mg/kg) every 3 days. The tumors were measured in the longest (L) and shortest (W) dimensions every four days for 28 days until the tumor volume was 1500 mm^3^. At the end of the experiments, the mice and excised tumors were photographed. All nude mice were euthanized by cervical dislocation after anesthesia with pentobarbital sodium.

### Hematoxylin-Eosin (HE) staining

Excised tumors were fixed in 4% formaldehyde, dehydrated, paraffin-embedded, and cut into 4-µm sections as previously described [28]. The sections were heated at 60°C for 2 h and stained with HE. The histology of the sections was analyzed using light microscopy (Nikon Eclipse E100, Tokyo, Japan).

### Immunohistochemistry (IHC)

After dewaxing, the sections were subjected to antigen retrieval by high-pressure heating and then exposed to 3% H_2_O_2_ for 10 min. After blocking, the sections were incubated with primary antibody for Lin28A (1:100; rabbit, Abcam, ab279647) or Lin28B (1:100; rabbit, Abcam, ab191881) overnight at 4°C, followed by incubation with goat anti-rat IgG (1:200; Abcam) at 37°C for 20 min. After incubation with diaminobenzidine (DAB), the sections were counterstained with hematoxylin. The slides were evaluated by light microscopy (Nikon Eclipse E100).

### TUNEL staining

Paraffin-embedded sections were stained using the TUNEL Apoptosis Assay Kit (Cat. No. 11,684,817,910, Roche, Basel, Swiss) according to the manufacturer’s instructions, and visualized by microscopy (Nikon, Japan). Five representative fields were selected, and the numbers of positive and negative cells were counted. The positive rate was calculated as follows: number of positive cells/ (number of positive cells + number of negative cells) × 100%.

### Statistical analysis

Data are presented as the mean ± the standard deviation (SD). Data analysis was performed using SPSS 21.0 software (SPSS, Inc.). Student’s t-test and one-way ANOVA were used to compare the differences for two groups and multiple groups, respectively. *P* < 0.05 indicated statistically significant differences.

## Results

### Identification of differentially expressed circRNAs (DECs) in exosomes derived from CAFs

**Fig. 1:**
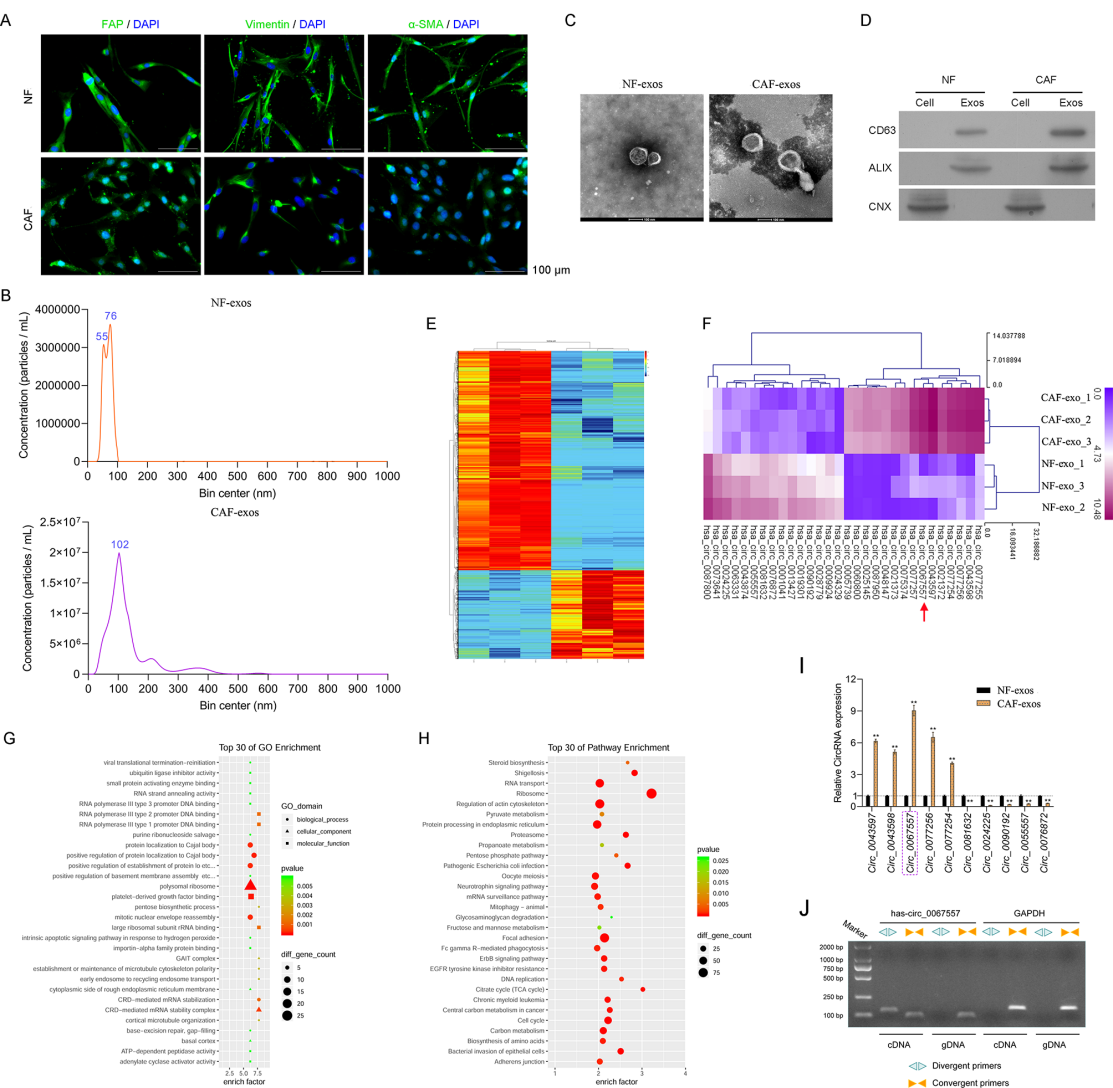
Identification of DECs in exosomes derived from NFs and CAFs. (**A**) IF staining of FAP, vimentin, and α-SMA in CAFs and NFs isolated from CRC and adjacent normal tissue, respectively; 20×magnification. (**B**) NanoSight particle size detection of exosomes isolated from CAF and NF culture medium. (**C**) The morphology of exosomes secreted by CAFs and NFs was observed by TEM. (**D**) Western blotting for CD63, ALIX, and CNX to identify exosomes. (**E**) Heat map of DECs in exosomes derived from NFs and CAFs confirmed by circular RNA microarray. The upregulated and downregulated DECs were selected by the criteria of fold of change (FC) > 1.5, *P* < 0.05. (**F**) Clustered heatmap of the top 15 upregulated and downregulated circRNAs in exosomes derived from NFs and CAFs. (**G**) GO analysis for DECs in exosomes secreted by CAFs and NFs. (**H**) KEGG analysis of DECs in exosomes from CAFs and NFs. KEGG analysis was based on www.kegg.jp/kegg/kegg1.html with the permission from the Kanehisa laboratory. (**I**) The expression of the top five upregulated and downregulated circRNAs in exosomes derived from NFs and CAFs was confirmed by RT-qPCR. (**J**) Expression of circ_0067557 in cDNA or gDNA was identified using divergent primers. Original gels were provided in Supplemental files

It has been reported that exosomes secreted by CAFs can drive tumor progression [29]. To explore the influence of CAF-derived exosomes on CRC progression, we isolated CAFs and NFs from CRC tumors and adjacent normal tissue. Successful isolation was confirmed by FAP, vimentin, and α-SMA staining (Fig. 1A). We next isolated exosomes from the culture medium of CAFs and NFs. The exosomes were uniformly dispersed, with diameter of 100 nm (Fig. 1B). Moreover, TEM showed that the isolated exosomes were circular particles with diameters of 100–200 nm and a clear double-membrane structure (Fig. 1C). Western blot analysis showed that exosomal markers CD63 and Alix were enriched in these samples, but the membrane organelle fraction marker calnexin (CNX) was absent (Fig. 1D). Thus, we successfully isolated NFs and CAFs and exosomes produced by these cells.

We identified DECs between NF- and CAF-derived exosomes using a circRNA microarray. Principal component analysis (PCA) showed that circRNAs with large differences could distinguish the NF-exosomes group and CAFs-exosomes group (Figure S1A). A matrix graph demonstrated that different groups of samples were correlated (Figure S1B,C). In Scatter and Volcano plots, red and blue represented the upregulated and downregulated circRNAs, respectively (Figure S1D, E). A heat map was created for the DECs from the exosomes derived from the NFs and CAFs (Fig. 1E). We used a clustered heatmap to identify top 15 upregulated and downregulated circRNAs in exosomes derived from the CAFs (Fig. 1F).

We predicted mRNAs that might be regulated by the DECs based on the complementary binding of miRNA with circRNA and mRNA 3’UTRs using Starbase, and the identified target mRNAs of the DECs were listed in Supplementary Table 2. GO and KEGG pathway analysis of these mRNAs showed that the top GO terms mainly included polysomal ribosomes, platelet-derived growth factor binding, mitotic nuclear envelope reassembly, and protein localization to Cajal bodies (Fig. 1G). KEGG pathway analysis identified multiple signaling pathways, including ribosome, regulation of the actin cytoskeleton, focal adhesion, and the cell cycle (Fig. 1H). These results suggest the potential correlation between the enriched functions and pathways of target mRNAs with CRC.

Moreover, we identified the top five upregulated circRNAs (circ_0043597, circ_0043598, circ_0067557, circ_0077256, and circ_0077254) and top five downregulated circRNAs (circ_0081632, circ_0024225, circ_0090192, circ_0055557, and circ_0076872) based on the biggest fold change from the exosomes derived from the CAFs. We focused on circ_0067557 because it was highly expressed in the exosomes derived from CAFs (Fig. 1I). Using divergent primers, the circ_0067557 target sequence was only amplified when cDNA, not gDNA, was used as the template, indicating that the detected circ_0067557 was not a by-product of PCR or gene rearrangement (Fig. 1J).

**Fig. 2:**
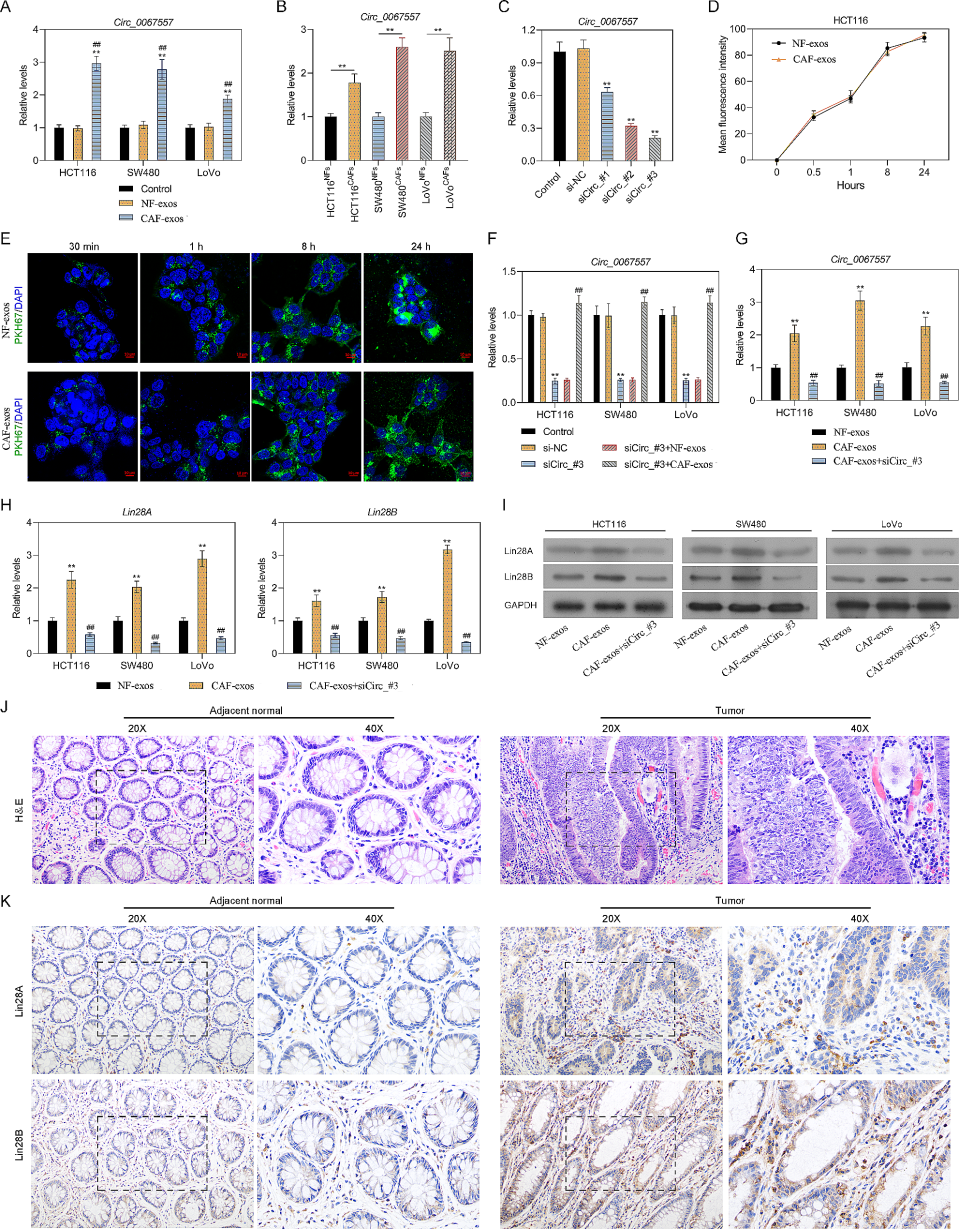
Circ_0067557 knockdown decreased Lin28A and Lin28B expression in CAF-derived exosomes. (**A**) RT-qPCR analysis of circ_0067557 in HCT116, SW480, and LoVo cells after co-culture with NFs and CAFs. (**B**) Circ_0067557 expression in HCT116, SW480, and LoVo cells was examined by RT-qPCR after exposure to exosomes from NFs and CAFs. (**C**) RT-qPCR analysis of circ_0067557 expression in HCT116 cells following circ_0067557 silencing. (**D-E**) Fluorescence of HCT116 cells treated with PKH67-labeled exosomes derived from NFs and CAFs. (**F**) CRC cells were transfected with si-circ_0067557 and then treated with exosomes derived from NFs and CAFs; circ_0067557 expression was measured by RT-qPCR. (**G**) Circ_0067557 expression in HCT116, SW480 and LoVo cells treated with si-circ_0067557 and CAF-derived exosomes was measured by RT-qPCR. Lin28A and Lin28B expression was evaluated by RT-qPCR (**H**) and Western blotting (**I**) in CRC cells treated with si-circ_0067557 and CAF-derived exosomes. (**J**) H&E staining of the sections of CRC tumors and adjacent normal tissues dissected from patients (*n* = 12). The glands were irregular with necrosis, interstitial fibrosis, infiltrating cells, and pathological mitosis in sections of CRC tumors. 20× and 40× magnification. (**K**) IHC showed higher expression of Lin28A and Lin28B in the sections of CRC tumors dissected from patients compared to adjacent normal tissues (*n* = 12). 20× and 40× magnification

According to the Starbase database, the targets of circ_0067557 could be Lin28A and Lin28B. Therefore, we examined whether changes in circ_0067557 levels in CAF-derived exosomes could affect Lin28A and Lin28B expression in CRC cells. Circ_0067557 expression was increased in HCT116, SW480, and LoVo CRC cells after co-culture with CAFs compared to co-culture with NFs (Fig. 2A). Similarly, circ_0067557 expression in CAF-derived exosomes was higher than in NF-derived exosomes (Fig. 2B). We next used three siRNAs to silence circ_0067557 in HCT116 cells and found that sicirc_0067557#3 reduced circ_0067557 levels most effectively (Fig. 2C). Moreover, we evaluated the uptake of PKH67-labeled exosomes derived from NFs and CAFs into HCT116 cells by IF. The green fluorescence intensity increased as time progressed and was the strongest at 24 h (Fig. 2D, E).

We treated circ_0067557-silenced HCT116, SW480, and LoVo cells with exosomes from NFs and CAFs. The exosomes from the CAFs notably abrogated the circ_0067557 downregulation mediated by sicirc_0067557#3 (Fig. 2F). Similarly, the silencing of circ_0067557 in HCT116, SW480 and LoVo cells attenuated the upregulation of circ_0067557 mediated by the CAF-derived exosomes (Fig. 2G). Furthermore, the elevated levels of Lin28A and Lin28B induced by CAF-derived exosomes could be dramatically reversed by circ_0067557 silencing in HCT116, SW480 and LoVo cells (Fig. 2H, I). Taken together, these findings indicated that exosomes derived from CAFs could increase Lin28A and Lin28B expression by upregulating circ_0067557.

We also compared CRC tumors with adjacent normal tissue. The glands in adjacent normal tissue were arranged regularly, with no nuclear atypia. In CRC tumors, the glands were irregular with necrosis, interstitial fibrosis, infiltrating cells, and pathological mitosis (Fig. 2J). IHC showed that Lin28A and Lin28B were significantly upregulated in CRC compared to the adjacent normal tissue (Fig. 2K).

### CAF-derived exosomes induced malignant characteristics of CRC cells by upregulating circ_0067557

**Fig. 3:**
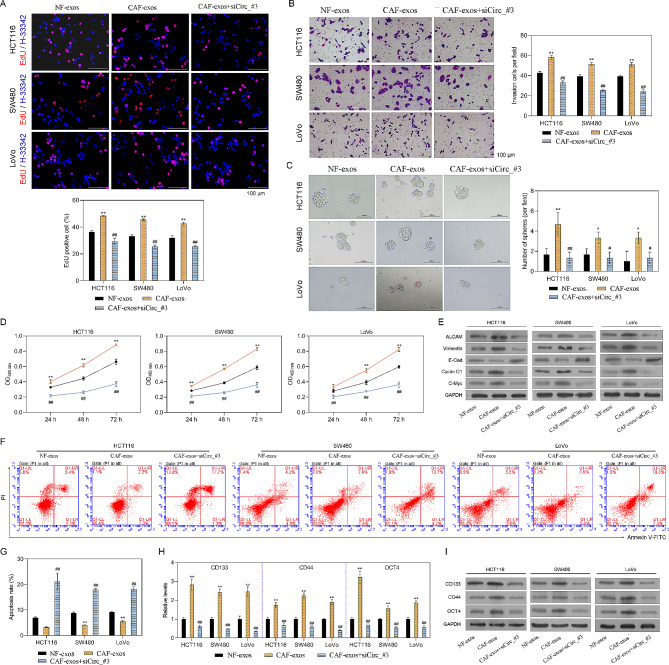
CAF-derived exosomes induced malignant phenotypes of CRC cells by upregulating circ_0067557. HCT116, SW480 and LoVo cells were treated with si-circ_0067557 and CAF-derived exosomes. (**A**) Cell proliferation was determined by EdU staining. (**B**) Cell invasion was detected by Transwell assay. (**C**) The number of spheres formed was analyzed by Sphere-forming assay. (**D**) Evaluation of 5-FU and L-OHP drug resistance at 24, 48, and 72 h using the CCK-8 assay. (**E**) Western blot analysis for ALCAM, vimentin, E-cadherin, cyclin D1, and c-Myc. (**F**) Apoptosis was measured by flow cytometry. (**G**) The apoptosis rate was calculated for each group. CD133, CD44, and OCT4 cell stemness markers were evaluated by RT-qPCR (**H**) and Western blotting (**I**)

We investigated the effects of CAF-derived exosomes and circ_0067557 silencing on malignant behaviors of CRC cells. HCT116, SW480 and LoVo cells were treated with CAF-exosomes with or without si-circ_0067557. Circ_0067557 silencing reduced CRC cell proliferation and sphere formation induced by CAF-derived exosomes (Fig. 3A, C). Moreover, CAF-derived exosomes promoted the migration of CRC cells, while circ_0067557 silencing reversed this effect (Figure S2). Circ_0067557 silencing also attenuated the invasion of CRC cells mediated by CAF-derived exosomes (Fig. 3B).

We also determined the effect of CAF-derived exosomes on the chemosensitivity of HCT116, SW480 and LoVo cells to 5-FU and L-OHP. CAF-derived exosomes made all three cell lines more resistant to the chemotherapeutic agents; however, the silencing of circ_0067557 attenuated this effect (Fig. 3D). Moreover, CAF-derived exosomes reduced apoptosis of CRC cell lines, which was markedly reversed by circ_0067557 silencing (Fig. 3F, G). The CAF-derived exosomes also caused the upregulation of ALCAM, vimentin, cyclin D1 and c-Myc and the downregulation of E-cadherin in HCT116, SW480 and LoVo cells (Fig. 3E). These effects were reversed by circ_0067557 silencing. Additionally, the silencing of circ_0067557 could reduce the upregulation of CD133, CD44 and OCT4 by CAF-derived exosomes in HCT116, SW480 and LoVo cells (Fig. 3H, I). Collectively, these results revealed that CAF-derived exosomes could increase the chemoresistance and malignant properties of CRC cells via targeting circ_0067557.

### Circ_0067557 regulated the phenotypes of HCT116 cells

**Fig. 4:**
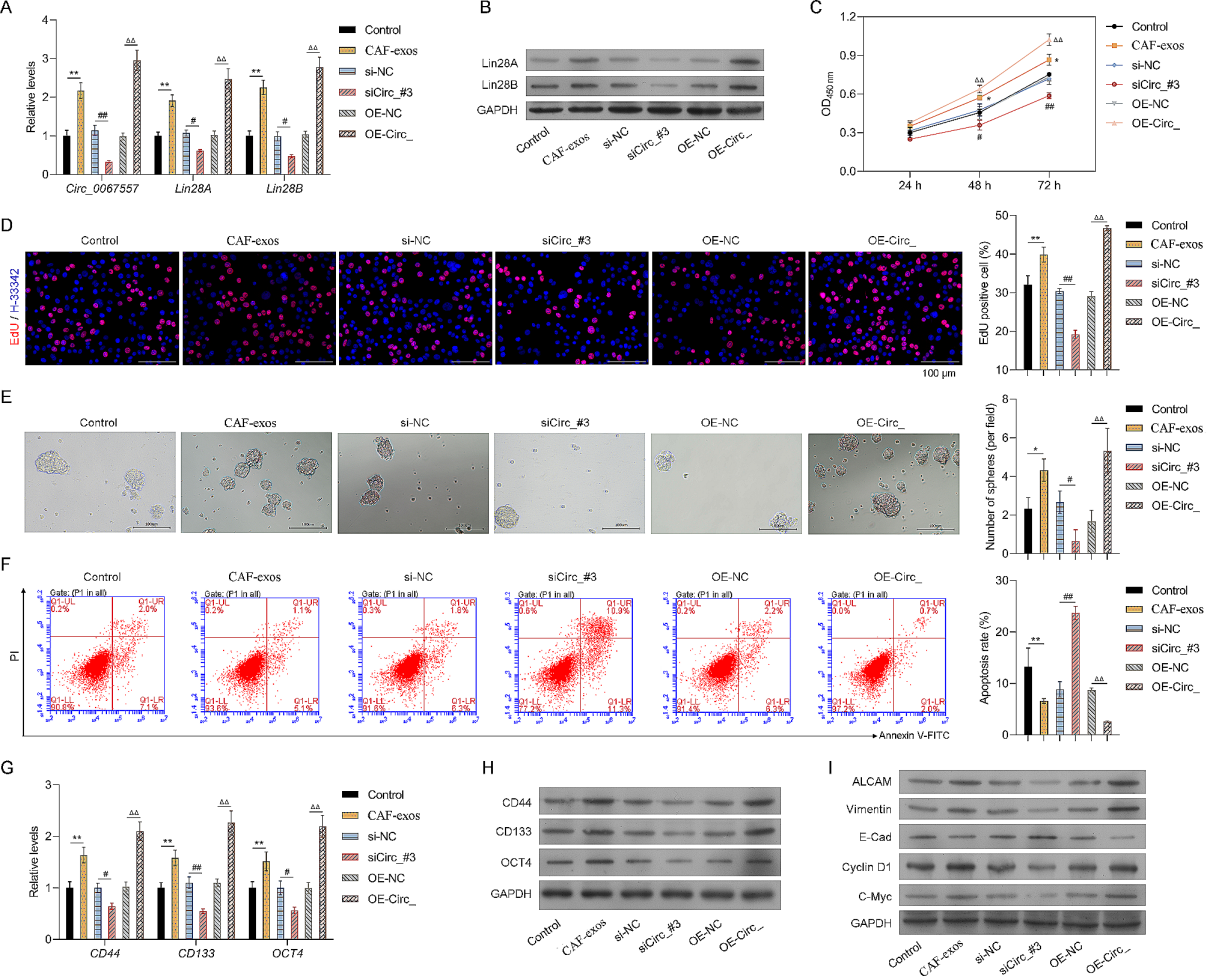
Circ_0067557 affected malignant phenotypes of HCT116 cells. Circ_0067557 was overexpressed or silenced in HCT116 cells. (**A**) Circ_0067557, Lin28A, and Lin28B expression levels were determined by RT-qPCR. (**B**) Lin28A and Lin28B expression levels were detected by Western blotting. (**C**) Drug resistance was determined by CCK-8 assay at 24, 48, and 72 h. (**D**) Proliferation was monitored by EdU staining. (**E**) Sphere-forming assay. (**F**) Apoptosis was examined by flow cytometry. CD133, CD44, and OCT4 expression levels were detected by Western blotting (**G**) and RT-qPCR (**H**). (**I**) The levels of EMT and cycle-related proteins were assessed by Western blotting

We next determined the effects of circ_0067557 on the proliferation and sphere formation of HCT116 cells. CAF-derived exosomes or circ_0067557 overexpression upregulated circ_0067557, Lin28A, and Lin28B levels (Fig. 4A, B) and facilitated proliferation and sphere formation of HCT116 cells (Fig. 4C-E), whereas circ_0067557 silencing downregulated their expression levels and inhibited cell proliferation and sphere formation. Consistent with the effects of CAF-derived exosomes, circ_0067557 overexpression reduced apoptosis (Fig. 4F) and promoted cancer stem cell (CSC) features (CD133, CD44, and OCT4 protein expression) (Fig. 4G, H) in HCT116 cells, while circ_0067557 knockdown caused the opposite effects. Moreover, circ_0067557 overexpression mimicked the effects of CAF-derived exosomes on epithelial-to-mesenchymal transition (EMT) of HCT116 cells, which was ameliorated by circ_0067557 silencing (Fig. 4I). Overall, these data suggested that circ_0067557 exhibited similar effects to CAF-derived exosomes on the proliferation, chemoresistance, EMT and CSC features of CRC cells.

### Overexpression of Lin28A and Lin28B antagonized the effects of circ_0067557 silencing on malignant behaviors of CRC cells

**Fig. 5:**
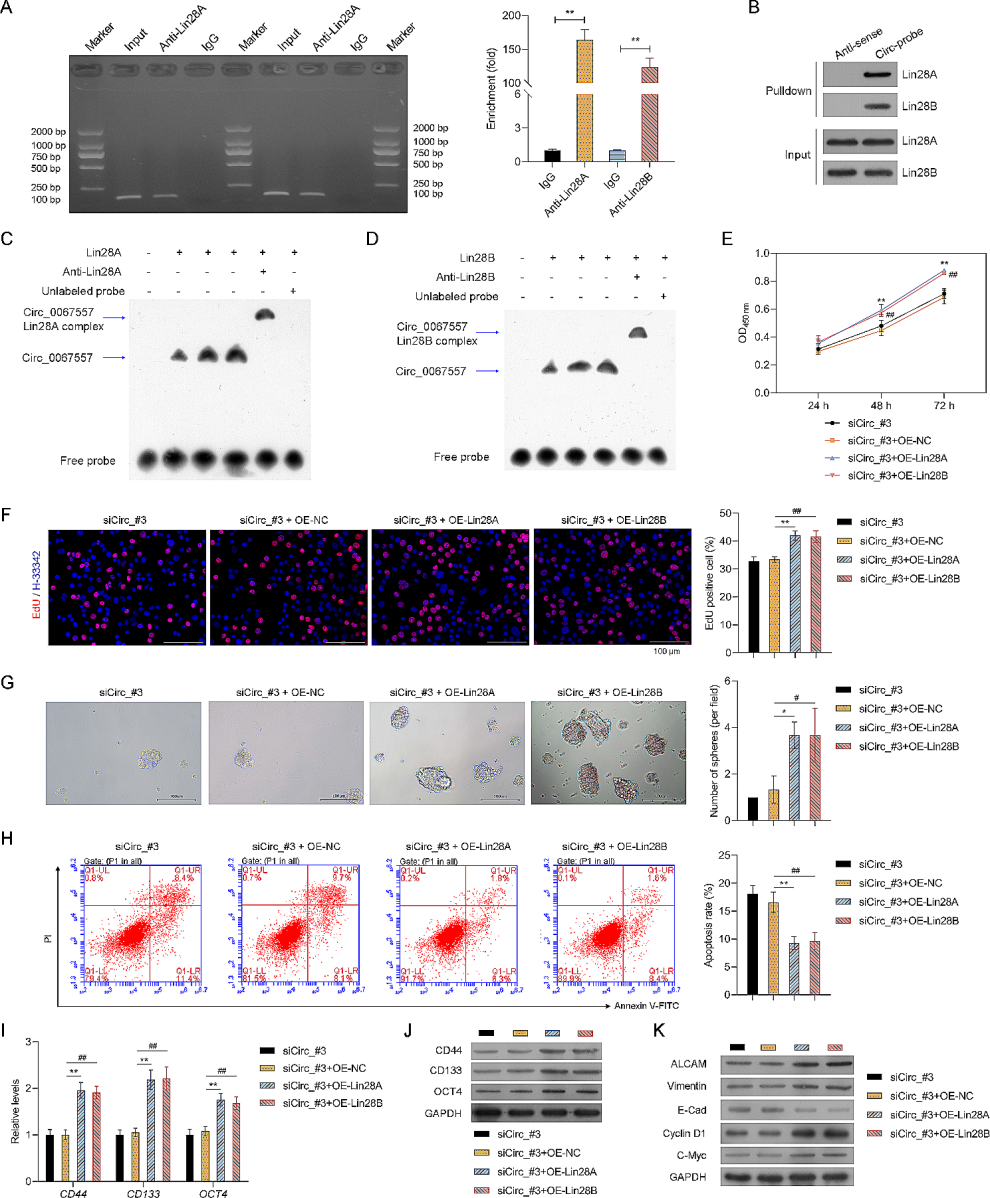
Overexpression of Lin28A and Lin28B attenuated the effects of circ_0067557 silencing on the malignant behaviors of CRC cells. (**A**) The interaction of circ_0067557 and Lin28A or Lin28B was monitored by the RIP assay. Original gels were provided in Supplemental files. (**B**) RNA-pulldown was used to evaluate the combination of circ_0067557 and Lin28A or Lin28B. The interaction between circ_0067557 and Lin28A (**C**) or Lin28B (**D**) was detected by EMSA assay. (**E**) Drug resistance (5-FU and L-OHP) was evaluated in circ_0067557-silenced HCT116 cells transfected with Lin28A- or Lin28B-overexpressing plasmids using the CCK-8 assay. (**F**) HCT116 proliferation was evaluated by EdU staining. (**G**) Sphere-forming assay. (**H**) Apoptosis was monitored by flow cytometry. CD133, CD44, and OCT4 expression levels were evaluated by RT-qPCR (**I**) and Western blotting (**J**). (**K**) EMT and cell cycle-related proteins were detected by Western blotting

Next, we investigated whether circ_0067557 could interact directly with Lin28A and Lin28B. The RIP results showed increased enrichment of circ_0067557 in the anti-Lin28A and anti-Lin28B compared to the IgG control group, and PCR confirmed that both Lin28A and Lin28B could bind to circ_0067557 (Fig. 5A). Similarly, biotinylated circ_0067557 could precipitate both circ_0067557/Lin28A and circ_0067557/Lin28B complexes, suggesting that circ_0067557 might regulate target genes by binding to Lin28A and Lin28B (Fig. 5B). EMSA data verified the interaction between circ_0067557 and Lin28A and Lin28B (Fig. 5C, D). Moreover, we conducted rescue experiments to overexpress Lin28A or Lin28B in HCT116 cells (Figure S3A, B) or HCT116 cells with circ_0067557 silencing (Figure S3C, D). We found that Lin28A or Lin28B overexpression increased chemoresistance, proliferation, and sphere formation of HCT116 cells with circ_0067557 silencing (Fig. 5E–G). Lin28A or Lin28B overexpression also decreased apoptosis in HCT116 cells with circ_0067557 silencing (Fig. 5H). Finally, the overexpression of Lin28A or Lin28B rescued EMT and CSC features of HCT116 cells that had been inhibited by circ_0067557 knockdown (Fig. 5I–K). Collectively, these results suggested that circ_0067557 knockdown may suppress malignant phenotypes of HCT116 cells by downregulating Lin28A and Lin28B.

### CAF-derived exosomes and circ_0067557 regulated the growth of CRC xenografts

**Fig. 6:**
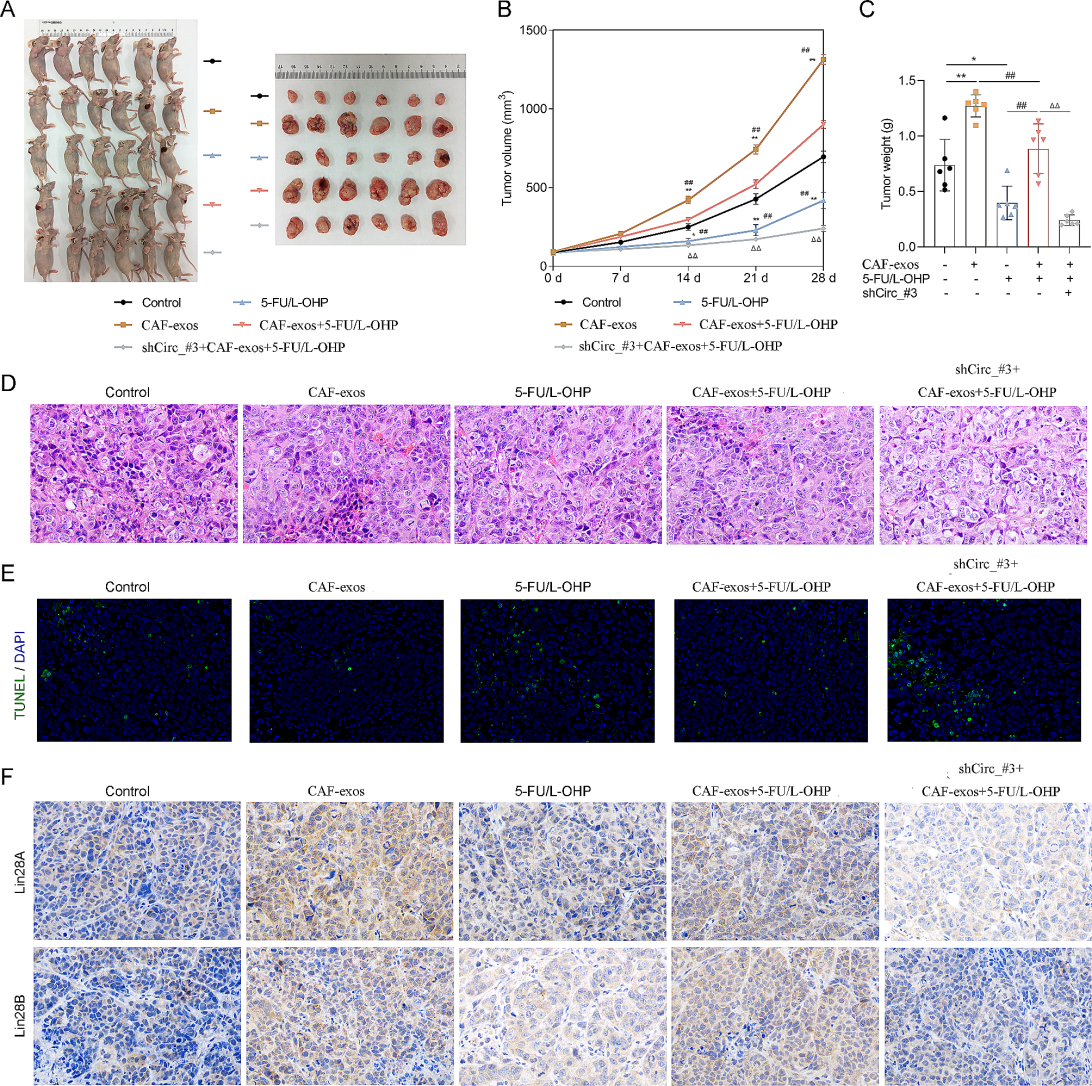
CAF-derived exosomes and circ_0067557 promoted tumor growth in CRC xenografts. Nude mice were injected with 100 µL normal saline in control group, 100 µL CAF-derived exosomes (10 µg, CAF-exos), 10 mg/kg 5-FU/L-OHP in experimental groups, respectively. (**A**) Tumor-bearing nude mice and excised tumors were photographed after four weeks. (**B**) The growth of the tumor was estimated by calculating the volume on days 7, 14, 21, and 28. (**C**) Tumor weights were measured at the end of four weeks. (**D**) Pathological changes in tumors were assessed by H&E staining. (**E**) Apoptosis was determined by TUNEL staining. (**F**) Lin28A and Lin28B expression was analyzed by IHC; 200× magnification

To investigate the role of CAF-derived exosomes and circ_0067557 in CRC tumor formation in vivo, nude mice were inoculated with HCT116 cells with or without circ_0067557 silencing. The mice were then treated with CAF-derived exosomes or/and 5-FU/L-OHP. Compared to the control group, tumor growth was dramatically enhanced by CAF-derived exosomes but was decreased by 5-FU and L-OHP. Moreover, tumor growth in mice treated with both CAF-derived exosomes and chemotherapy was less than in mice treated with only CAF-derived exosomes; tumors in mice treated with only 5-FU and L-OHP were smaller than those in mice receiving CAF-derived exosomes and 5-FU and L-OHP. The least tumor growth was observed in mice treated with shCirc_#3, CAF-derived exosomes, and 5-FU and L-OHP (Fig. 6A–C).

H&E staining showed that the tumors from control group were nodular, evenly distributed, and densely arranged. CAF-derived exosomes promoted tumor cell proliferation and inflammatory cell infiltration. After 5-FU/L-OHP treatment, tumor cells became less and showed nuclear condensation, fragmentation and dissolution, cytoplasm coagulation and necrosis, interstitial edema. Compared with CAF_exos + 5-FU/L-OHP group, the above pathological changes were further aggravated in shCirc #3 + CAF_exos + 5-FU/L-OHP group (Fig. 6D). CAF-derived exosomes reduced the number of TUNEL-positive cells in CRC tumors, while 5-FU/L-OHP increased the number of TUNEL-positive cells in CRC tumors, which was further increased by circ_0067557 silencing (Fig. 6E). IHC showed that CAF-derived exosomes could upregulate Lin28A and Lin28B in CRC tumors, while 5-FU and L-OHP could downregulate Lin28A and Lin28B in CRC tumors or CAF-derived exosome-treated CRC tumors. Circ_0067557 silencing reduced Lin28A and Lin28B expression levels in CRC tumors (Fig. 6F). These findings demonstrated the role of CAF-derived exosomes and circ_0067557 in regulating tumor growth and Lin28A and Lin28B expression in CRC xenografts.

## Discussion

Exosomes represent key modes of intercellular communication to transport the active substances and are crucial in tumor metastasis, drug resistance, immune response, and protein metabolism [30]. CAF in the TME play an indispensable role in stimulating cancer cells to undergo CSC transformation in prostate cancer and lung cancer, and CAF-derived exosome enhance colon stem cell resistance to 5-fluorouracil by activating Wnt pathway [31]. In this study, we successfully isolated NFs and CAFs from adjacent normal and CRC tissue, respectively, and further isolated exosomes from these two cell types. In addition, CAF-derived exosomes could endow colon cancer cells with EMT and CSC-like phenotypes, which are involved in chemoresistance.

CircRNAs regulate gene transcription or splicing, translation and epigenetics by competitively binding miRNAs and interacting with RNA-binding proteins [32]. Exosome-derived circRNAs are associated with pathological features and poor prognosis of patients and provide new targets for tumor diagnosis and treatment [33]. In current study, we used high-throughput sequencing to screen DECs between exosomes from NFs and those from CAFs, and found that the expression of circ_0067557 was relatively higher, and it has similar effects on CRC cells to CAF-derived exosomes. Our mechanistic analysis showed that circ_0067557 can play a sponge role in binding to its targets Lin28A and Lin28B, and play an important role in tumor progression and chemoresistance of CRC. Functionally, we found that silencing circ_0067557 by targeting Lin28A and Lin28B reversed the promoting effects of CAF-derived exosomes on CRC malignant phenotypes, including proliferation, migration, invasion, sphere-forming ability, chemoresistance, EMT, CSC characteristics and apoptosis. We also found that CAF-derived exosomes could promote the growth of colorectal cancer xenografts, while chemotherapeutic drugs could inhibite their growth by regulating circ_0067557. These results suggest that circ_0067557 is a potential target for CRC therapy to improve the prognosis of patients as well as their quality of life.

To our knowledge, this is the first report on the role of circ_0067557 in cancer. Only one study reported the involvement of circ_0067557 in preeclampsia, and the results showed that circ_0067557 may function as ceRNAs affecting PI3K-Akt signaling pathway [34]. Therefore, it would be interesting to investigate whether circ_0067557 also regulates PI3K-Akt signaling pathway in CRC.

Lin28 is a member of the RNA binding protein (RBP) family with a highly conserved and unique RNA binding motif, and Lin28A and Lin28B are two isotypes of this gene family [35]. Recent study confirmed that Lin28 is a key regulator of induced pluripotent stem cells (iPS) [36]. High Lin28 expression has been observed in multiple malignant tumors, including rhabdomyosarcoma, prostate cancer, and breast cancer [37]. However, the role of two subtypes of Lin28, Lin28A and Lin28B, in CRC development has rarely been reported. Therefore, it is important to understand RBP-RNA complexes, which might provide new strategies for CRC therapy [38].

In present study, we found that Lin28A and Lin28B were highly expressed in the tissues of CAC mice and CRC patients. We demonstrated for the first time that Lin28A and Lin28B could be targeted by circ_0067557, and Lin28A or Lin28B overexpression reversed the effects of circ_0067557 knockdown on CRC progression. Circ_0067557 silencing could downregulate Lin28A and Lin28B in CAF-derived exosomes and CRC xenograft tumors. However, this study has some limitations. Small molecular inhibitor of Lin28 has been identified and could be used to confirm that circ_0067557 regulates Lin28 to execute the effects on CRC progression because other targets of circ_0067557 in addition to Lin28 may be involved in CRC. Furthermore, other circRNAs differentially expressed in CAF-derived exosomes need to be explored in the context of CRC progression. A larger panel of CRC cell lines should be evaluated to verify the role of CAF-derived exosome-circ_0067557-Lin28A/B axis in CRC.

## Conclusions

Circ_0067557 expression in exosomes from CAFs was higher than in exosomes from NFs. Interfering with circ_0067557 expression by targeting Lin28A and Lin28B reversed the effects of CAF-derived exosomes to promote CRC malignant phenotypes, including proliferation, migration, invasion, sphere-forming ability, chemoresistance, EMT, CSC characteristics and apoptosis. Therefore, exosomal circ_0067557 is a potential therapeutic target for CRC to improve the prognosis of patients.

### Electronic supplementary material

Below is the link to the electronic supplementary material.

**Supplementary Material 1: Supplementary Figure 1. The expression profile of DECs in exosomes derived from NFs and CAFs.** (**A**) The clustering of samples was visualized via PCA analysis. g T indicated genome for tumor tissues, gN indicated genome for normal tissues. The plot axis indicated the normalized values. (**B**) Thehierarchical clusteringplot for ex osomes derived from NFs and CAFs. (**C**) Matrix graph forsample correlation analysis. (**D**) Scatter plot of DECs in exosomes derived from NFs and CAFs. (**E**) Volcan o plot for the DECs in exosomes derived from NFs and CAFs. Red represent the upr egulated DECs; blue represent the downregulated DECs. **Supplementary Figure 2. CAF-derived exosomes enhanced CRC cell migration by upr egulating circ_0067557.** HCT116, SW480 and LoVo cells were treated with CAF-deriv ed exosomes and si-circ_0067557 and cell migration was evaluated by wound healing assay. **Supplementary Figure 3. Overexpression of Lin28A and Lin28B in HCT116 cells.** Overexpression of Lin28A or Lin28B in HCT116 cells was determined by Western blotting (**A**) and RT-qPCR (**B**). Lin28A and Lin28B expression in circ_0067557-silenced HCT116 cells following transfection with Lin28A- or Lin28B-overexpressing plasmid was assessed by Western blotting (**C**) and RT-qPCR (**D**). **Supplementary Table 1. The primers used in this study**

**Supplementary Material 2: Supplementary Table 2.** The list of identified target mRNAs of the DECs

**Supplementary Material 3: **Original blots for Western blot analysis and original gels for agarose electrophoresis

**Supplementary Material 4: **The permission of KEGG

## Data Availability

All data generated or analyzed during this study are included in this published article and its supplementary information files.

## Abbreviations

TME: tumor microenvironment
CAFs: cancer-associated fibroblasts
CRC: colorectal cancer
circRNA: circular RNA
NFs: normal fibroblasts
RT-qPCR: real-time quantitative PCR
RIP: RNA immunoprecipitation
EMSA: electrophoretic mobility shift assay
EMT: epithelial-meseenchyal transition
CSC: cancer stem cell
EdU: 5-Ethynyl-2^,^-deoxyuridine
mRNAs: messenger RNAs
miRNAs: microRNAs
circRNAs: circular RNAs
lncRNAs: long non-coding RNAs
DECs: differentially expressed circRNAs
FBS: fetal bovine serum
5-FU: 5-Fluorouracil
L-OHP: oxaliplatin
BSA: bovine serum albumin
TEM: transmission electron microscopy
DLS: dynamic light scattering
ECL: enhanced chemiluminescence
PFA: paraformaldehyde
HE: Hematoxylin-Eosin
IHC: immunohistochemistry
DAB: diaminobenzidine
SD: standard deviation
PCA: principal component analysis
RBP: RNA binding protein
iPS: induced pluripotent stem cells
