# Supplementary material for: Exosomes derived from cancer-associated fibroblasts promote tumorigenesis, metastasis and chemoresistance of colorectal cancer by upregulating circ_0067557 to target Lin28
Source: BMC Cancer. 2024 Jan 12;24:64. doi: 10.1186/s12885-023-11791-5 (PMC10785442; doi:10.1186/s12885-023-11791-5)
Supplement: Supplementary file 1 — Supplementary Material 1: Supplementary Figure 1. The expression profile of DECs in exosomes derived from NFs and CAFs. (A) The clustering of samples was visualized via PCA analysis. g T indicated genome for tumor tissues, gN indicated genome for normal tissues. The plot axis indicated the normalized values. (B) Thehierarchical clusteringplot for ex osomes derived from NFs and CAFs. (C) Matrix graph forsample correlation analysis. (D) Scatter plot of DECs in exosomes derived from NFs and CAFs. (E) Volcan o plot for the DECs in exosomes derived from NFs and CAFs. Red represent the upr egulated DECs; blue represent the downregulated DECs. Supplementary Figure 2. CAF-derived exosomes enhanced CRC cell migration by upr egulating circ_0067557. HCT116, SW480 and LoVo cells were treated with CAF-deriv ed exosomes and si-circ_0067557 and cell migration was evaluated by wound healing assay. Supplementary Figure 3. Overexpression of Lin28A and Lin28B in HCT116 cells. Overexpression of Lin28A or Lin28B in HCT116 cells was determined by Western blotting (A) and RT-qPCR (B). Lin28A and Lin28B expression in circ_0067557-silenced HCT116 cells following transfection with Lin28A- or Lin28B-overexpressing plasmid was assessed by Western blotting (C) and RT-qPCR (D). Supplementary Table 1. The primers used in this study [file 12885_2023_11791_MOESM1_ESM.doc]

**
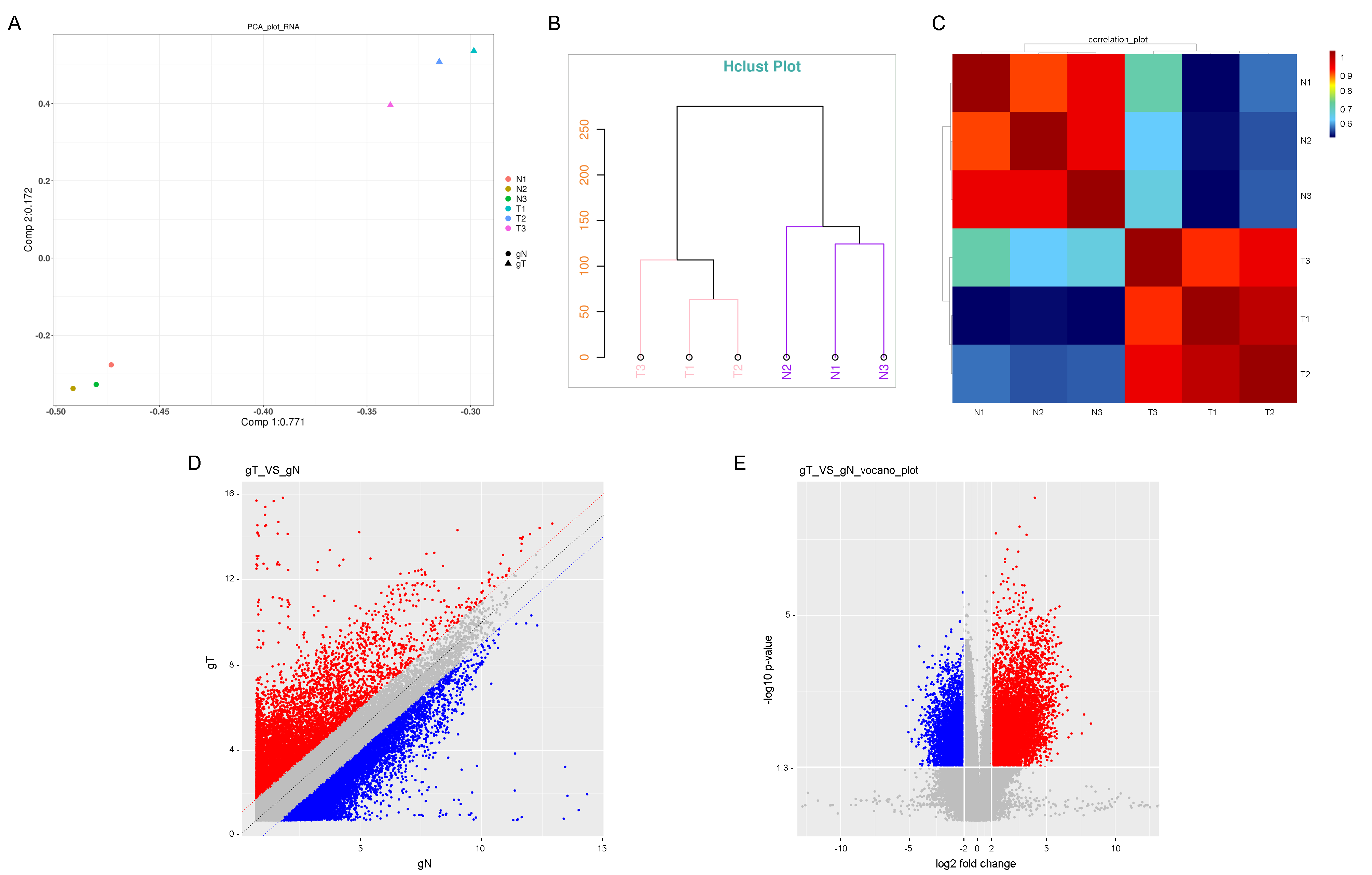
**

**Supplementary Figure 1. The expression profile of DECs in exosomes derived fro m NFs and CAFs.**(**A**) The clustering of samples was visualized via PCA analysis. g T indicated genome for tumor tissues, gN indicated genome for normal tissues. The plot axis indicated the normalized values. (**B**) Thehierarchical clusteringplot for ex osomes derived from NFs and CAFs. (**C**) Matrix graph forsample correlation analy sis. (**D**) Scatter plot ofDECs in exosomes derived from NFs and CAFs.(**E**)Volcan o plot for the DECs in exosomes derived from NFs and CAFs.Red represent the upr egulated DECs; blue represent the downregulated DECs.

**
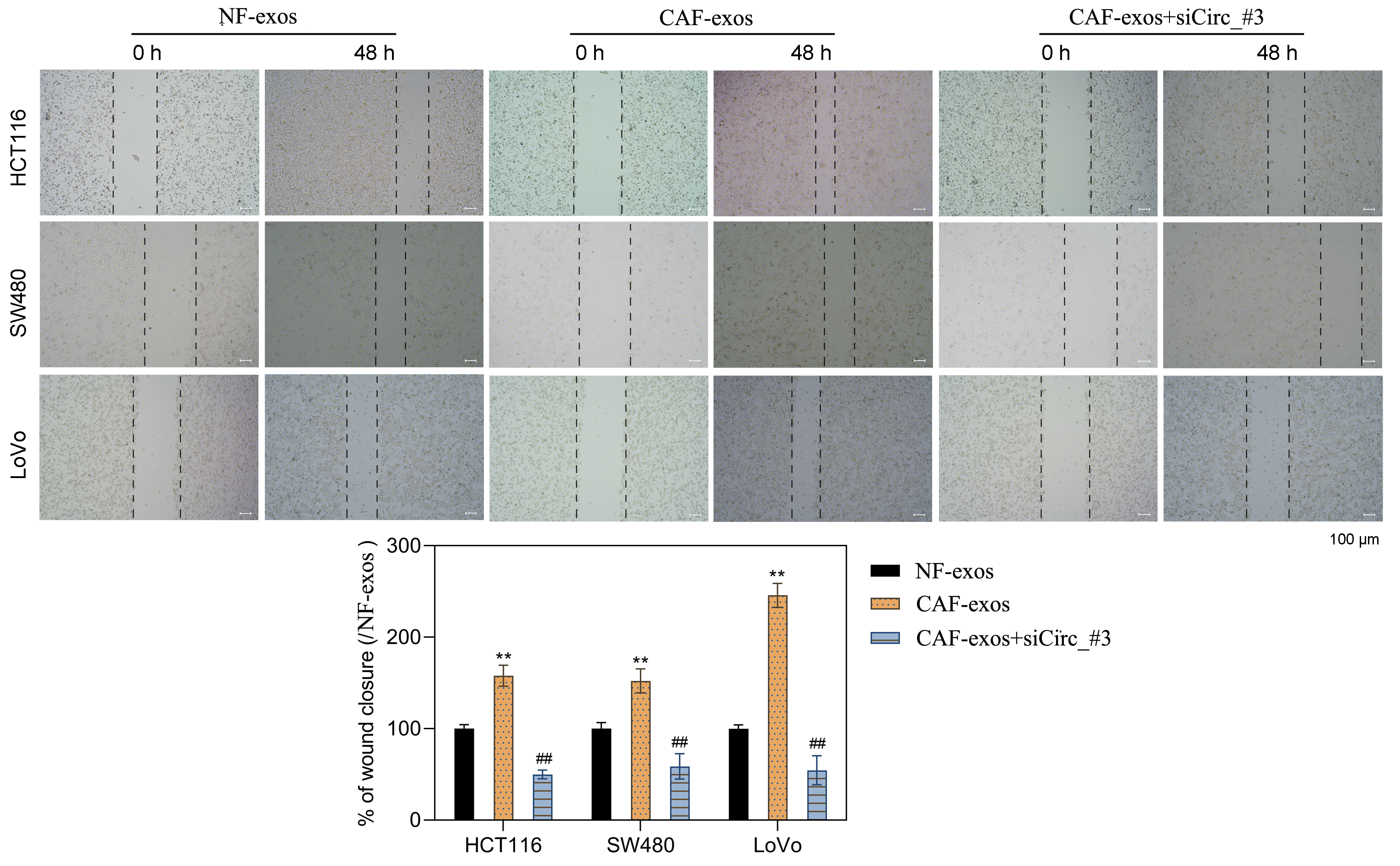
**

**Supplementary Figure 2. CAF-derived exosomes enhanced CRC cell migration by upregulating circ_0067557.** HCT116, SW480 and LoVo cells were treated with CAF-derived exosomes and si-circ_0067557 and cell migration was evaluated by wound healing assay.

**
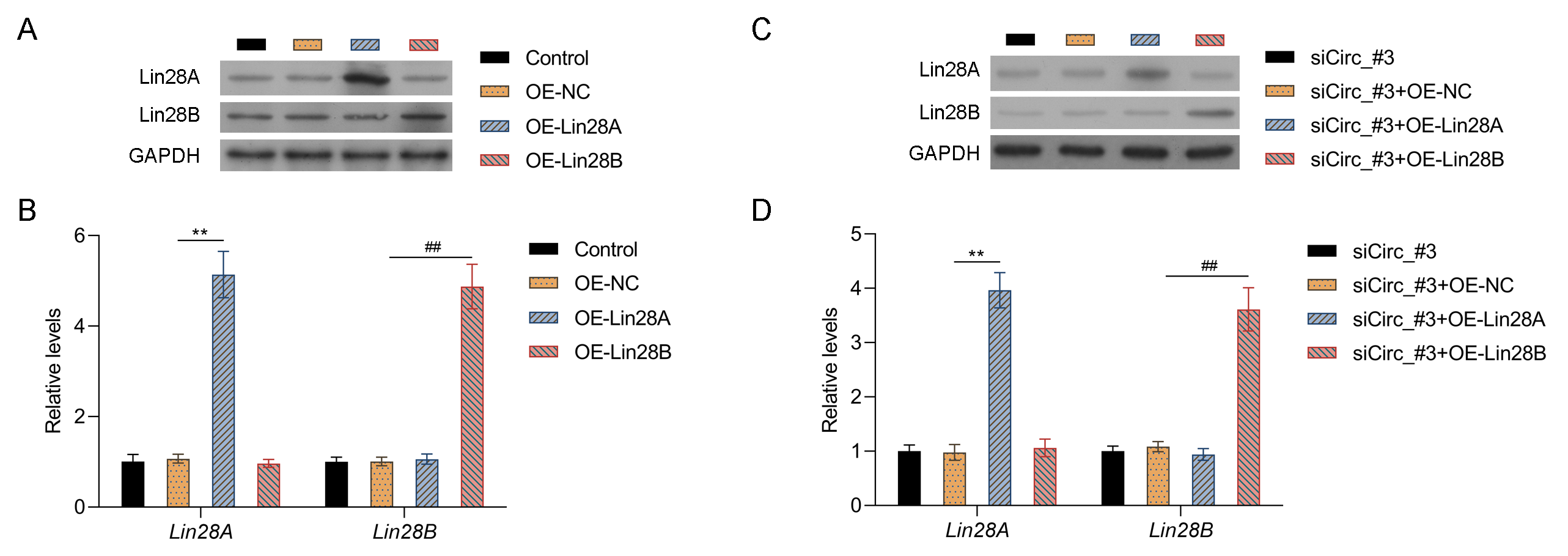
**

**Supplementary Figure 3.** **Overexpression of Lin28A and Lin28B in HCT116 cells.** Overexpression of Lin28A or Lin28B in HCT116 cells was determined by Western blotting (**A**) and RT-qPCR (**B**). Lin28A and Lin28B expression in circ_0067557-silenced HCT116 cells following transfection with Lin28A- or Lin28B-overexpressing plasmid was assessed by Western blotting (**C**) and RT-qPCR (**D**).

**Supplementary Table 1. The primers used in this study.**

| Gene | Sequences |
| --- | --- |
| hsa_circ_0043597 | F:CTTGGCCCCTCAGCGTACTGA  R:GTGTCTTCTGGGTAGAGGGAT |
| hsa_circ_0043598 | F:GCGCAGAGCCTGTTCCGTCTC  R:CTTTGGAGGGTGTCTTCTGGG |
| hsa_circ_0067557 | F:AAGTGATCTGTGTTCAAAACG  R:CCTGCTGATTCCTTGGGACAA |
| hsa_circ_0077256 | F:TGGCTACTCGTCCACACTCAG  R:TCTGCCAACAGTGAGGGTCGC |
| hsa_circ_0077254 | F:CTGGCTACTCGTCCACACTCA  R:CATGTAATTTTGGATTATGTT |
| hsa_circ_0081632 | F:GCACCGTCATCAACTGGTTCC  R:GCTGGCGCCCTGGCTGTACTC |
| hsa_circ_0024225 | F:GAGGAAATTAACTATCTGTAC  R:GTATGCATTTTCAAAAGCTGT |
| hsa_circ_0090192 | F:CGTCTTCTAGTGGTGAGTCTC  R:CTTCGACACATCACCGTGGTG |
| hsa_circ_0055557 | F:ATCTAAAGTGCTGATAATTAC  R:GGATAGTGACGAAATGGAACA |
| hsa_circ_0076872 | F:GCGCATCTGCGGCATTTCCTG  R:GGGTTATGCTGGAATTCGGTG |
| Lin28A | F:TGCGGGCATCTGTAAGTGG  R:GGAACCCTTCCATGTGCAG |
| LIN28B | F:CATCTCCATGATAAACCGAGAGG  R:GTTACCCGTATTGACTCAAGGC |
| CD133 | F:AGTCGGAAACTGGCAGATAGC  R:GGTAGTGTTGTACTGGGCCAAT |
| CD44 | F:CTGCCGCTTTGCAGGTGTA  R:CATTGTGGGCAAGGTGCTATT |
| OCT4 | F:CTTGAATCCCGAATGGAAAGGG  R：GTGTATATCCCAGGGTGATCCTC |
