# Supplementary material for: Exosomes derived from cancer-associated fibroblasts promote tumorigenesis, metastasis and chemoresistance of colorectal cancer by upregulating circ_0067557 to target Lin28
Source: BMC Cancer. 2024 Jan 12;24:64. doi: 10.1186/s12885-023-11791-5 (PMC10785442; doi:10.1186/s12885-023-11791-5)
Supplement: Supplementary file 3 — Supplementary Material 3: Original blots for Western blot analysis and original gels for agarose electrophoresis [file 12885_2023_11791_MOESM3_ESM.pdf]

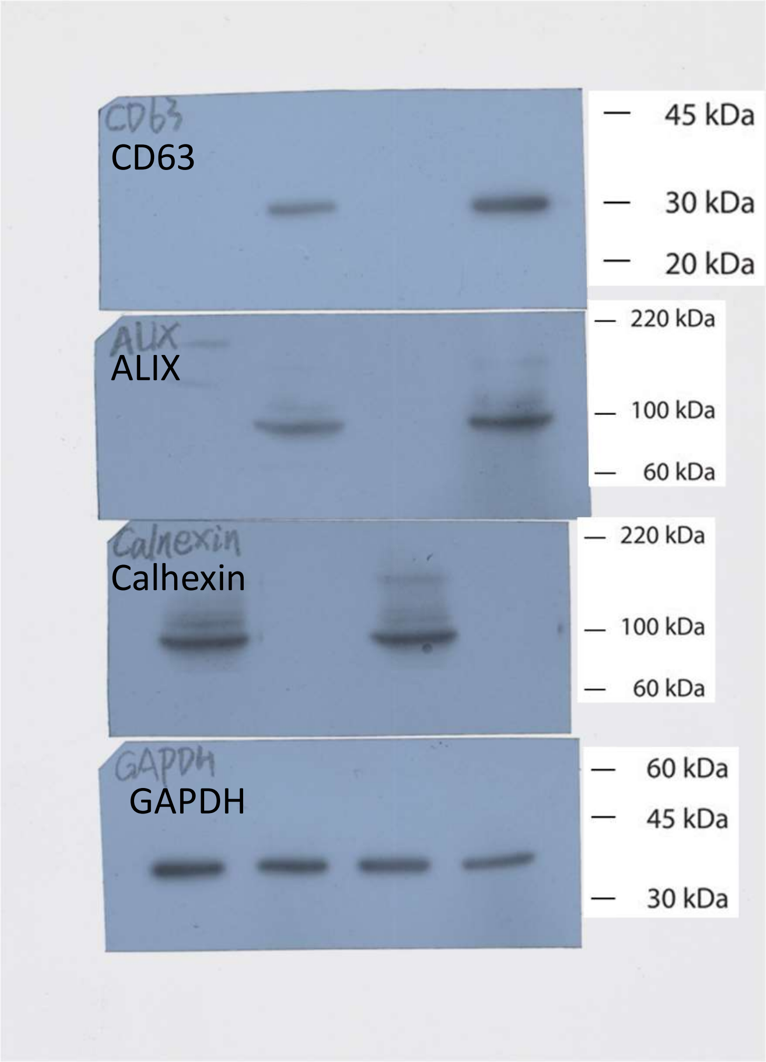

Original blots for Fig.1D

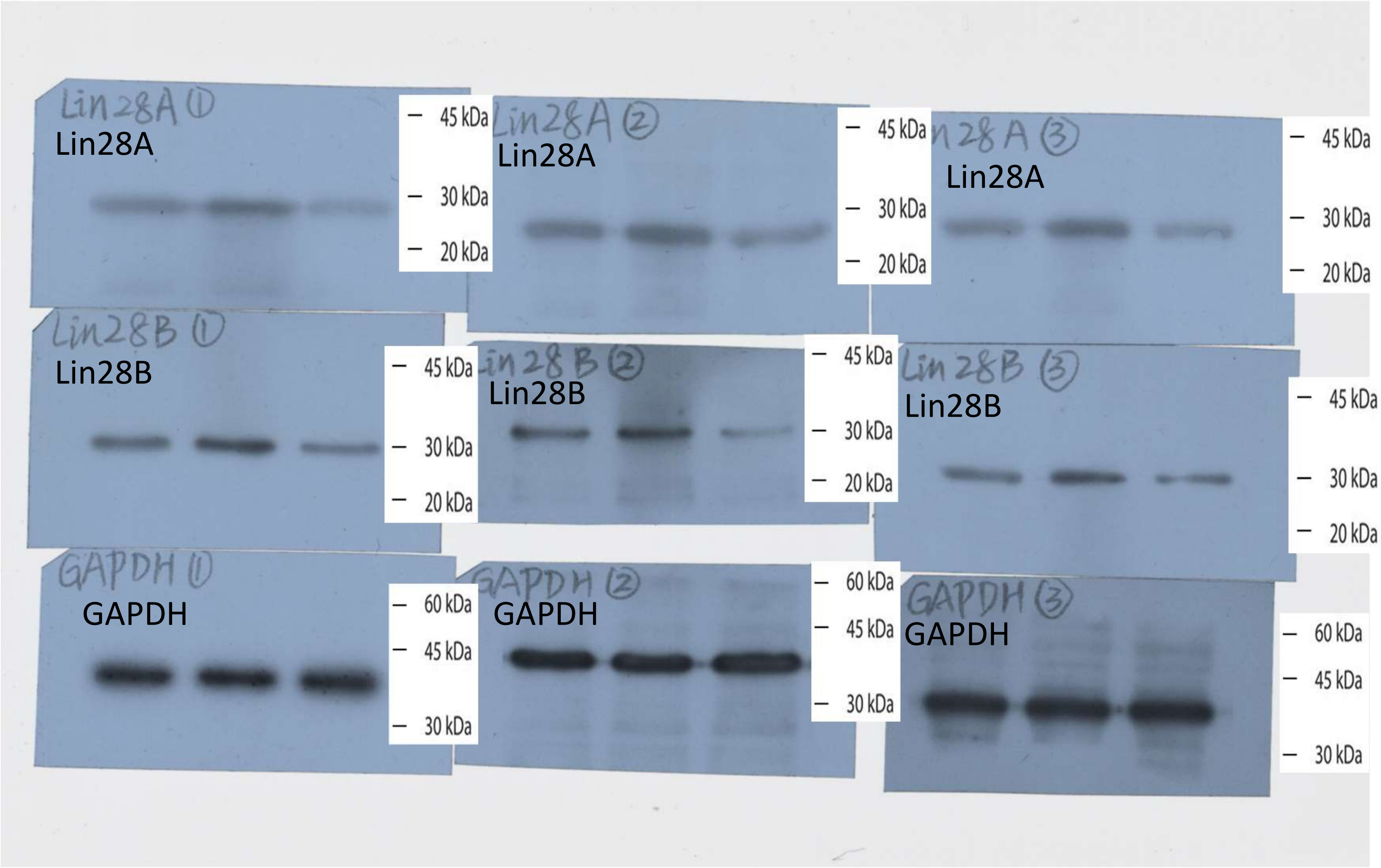

Original blots for Fig. 2I

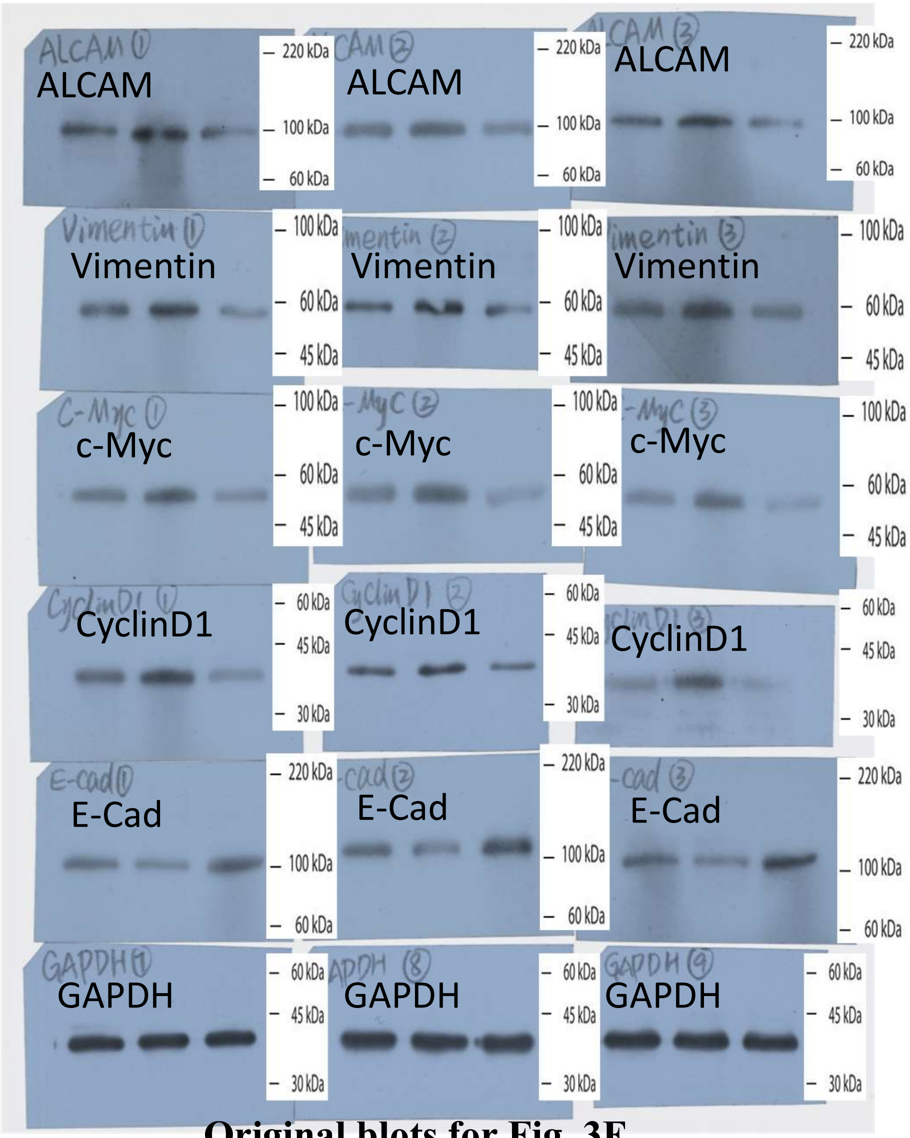

Original blots for Fig. 3E

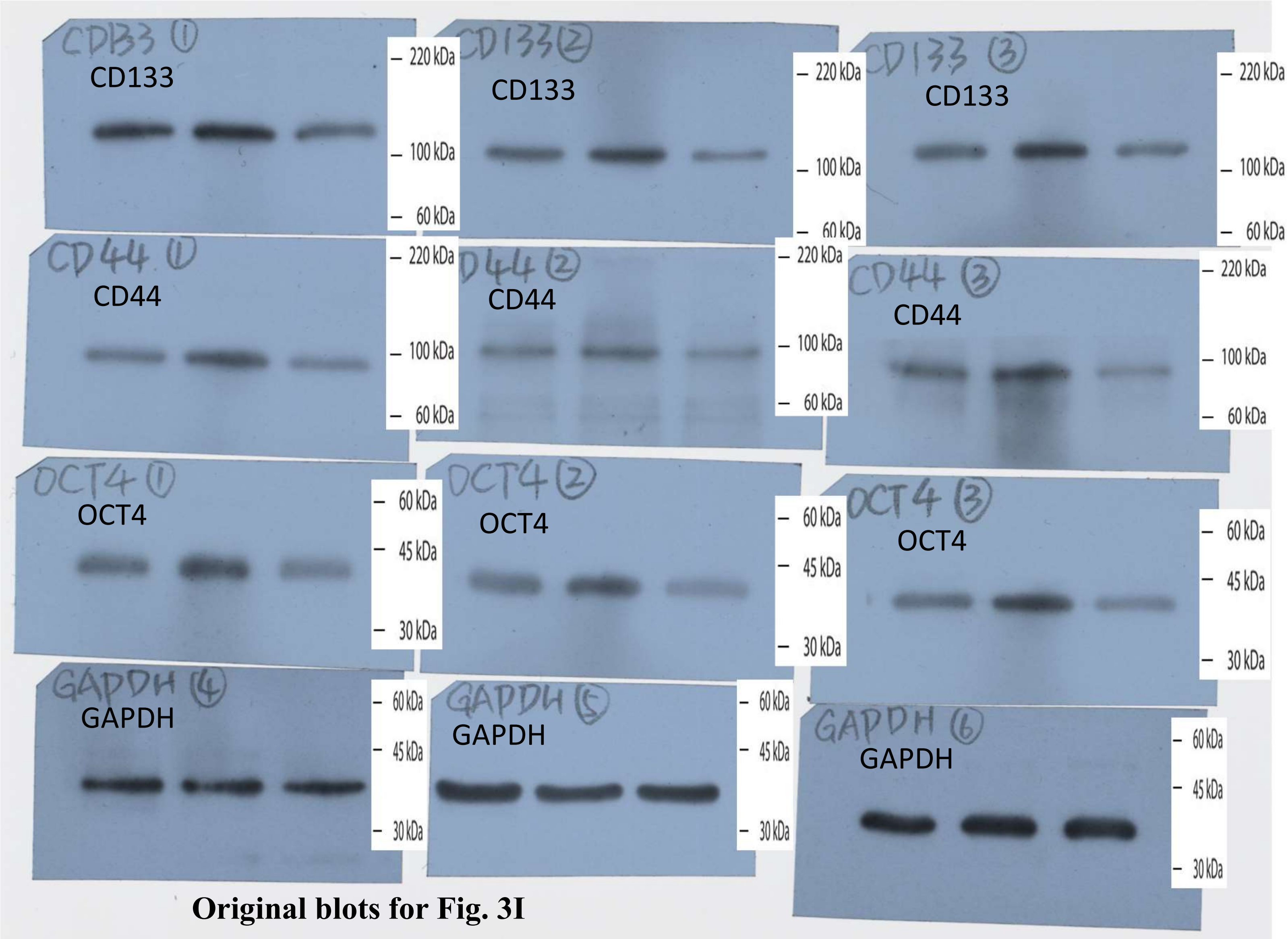

Original blots for Fig. 3I

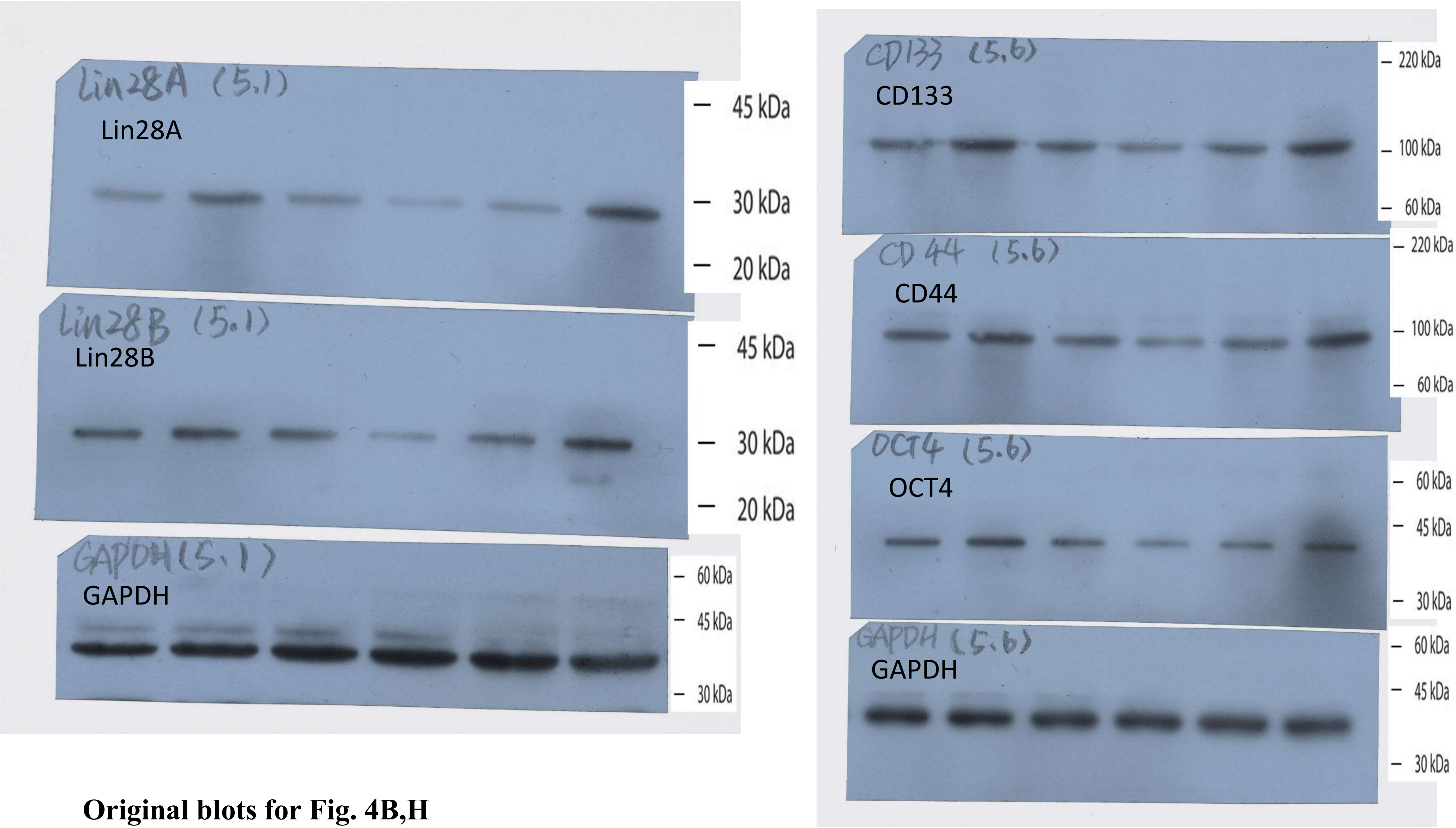

Original blots for Fig. 4B,H

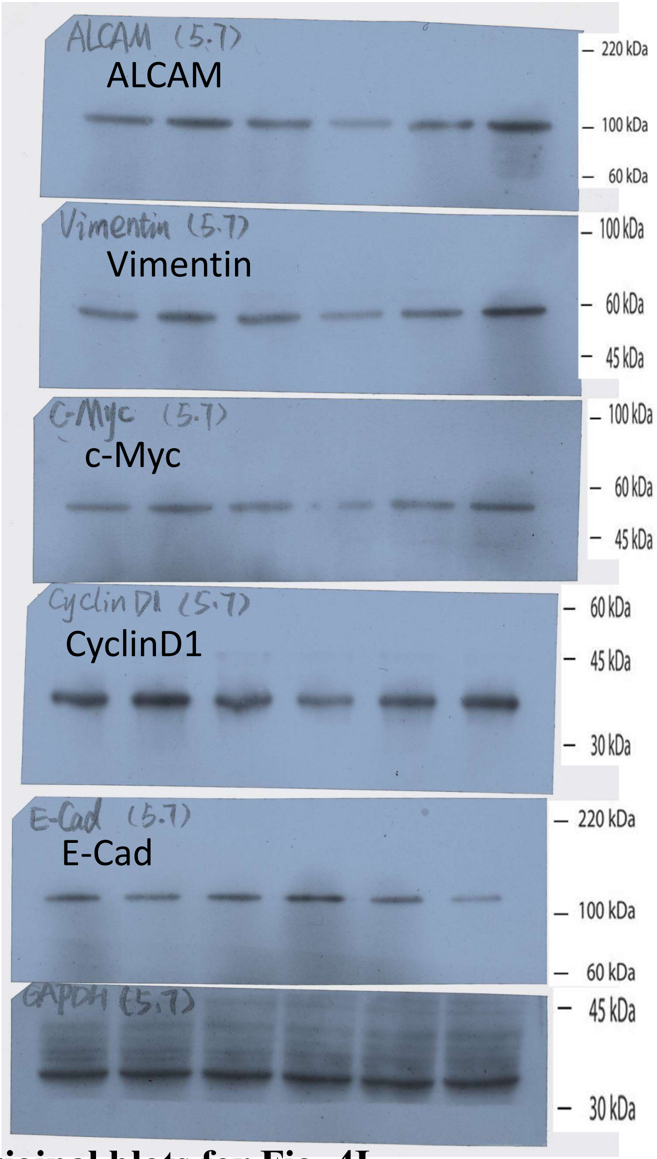

Original blots for Fig. 4I

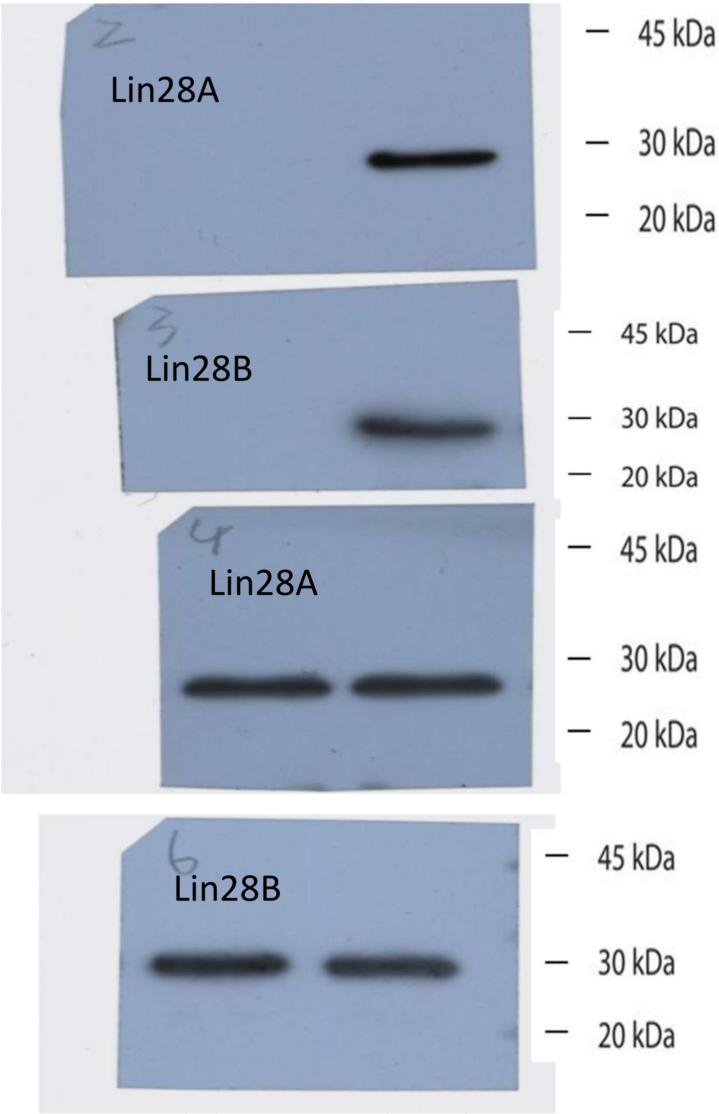

Original blots for Fig. 5B

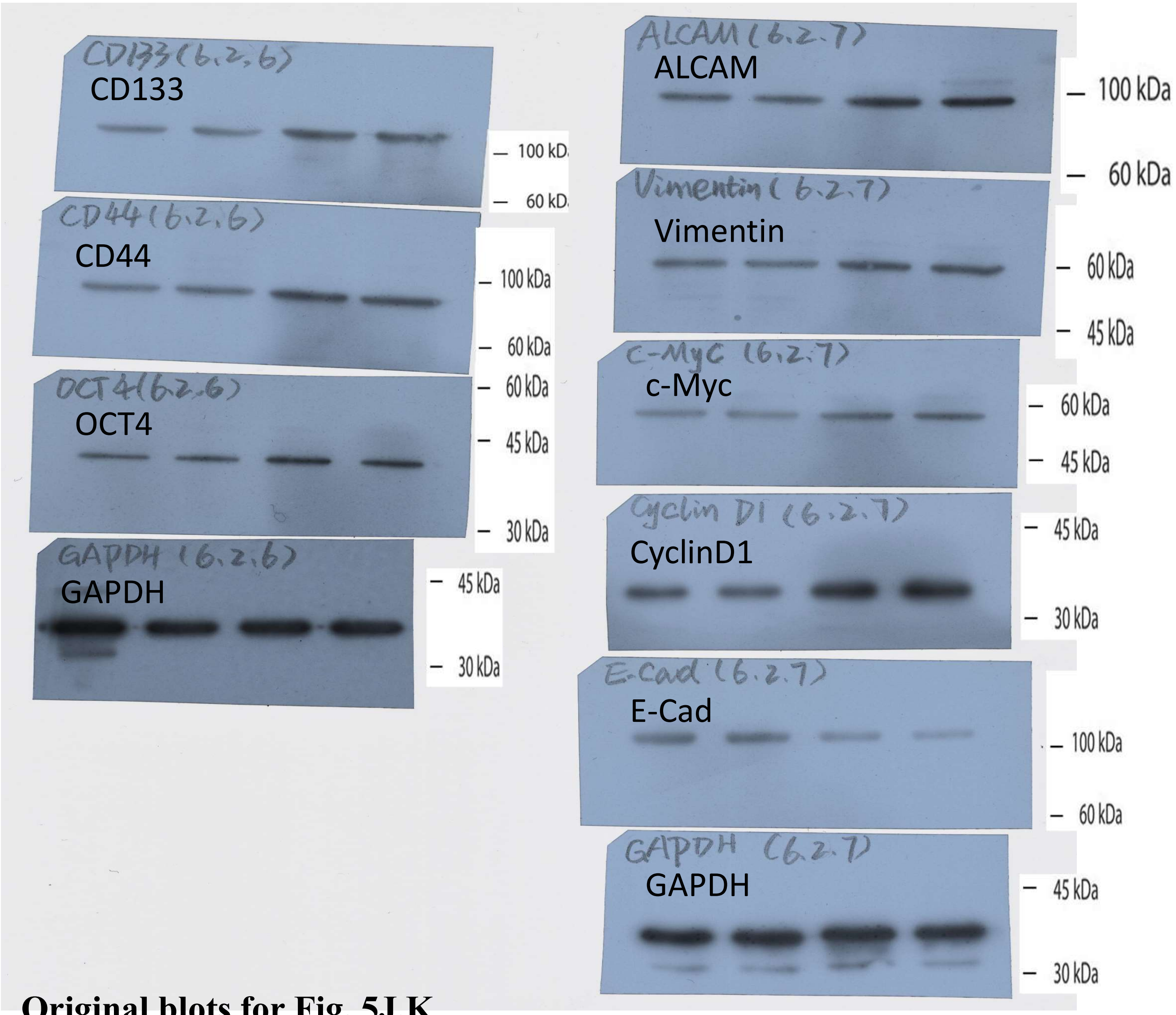

Original blots for Fig. 5J,K

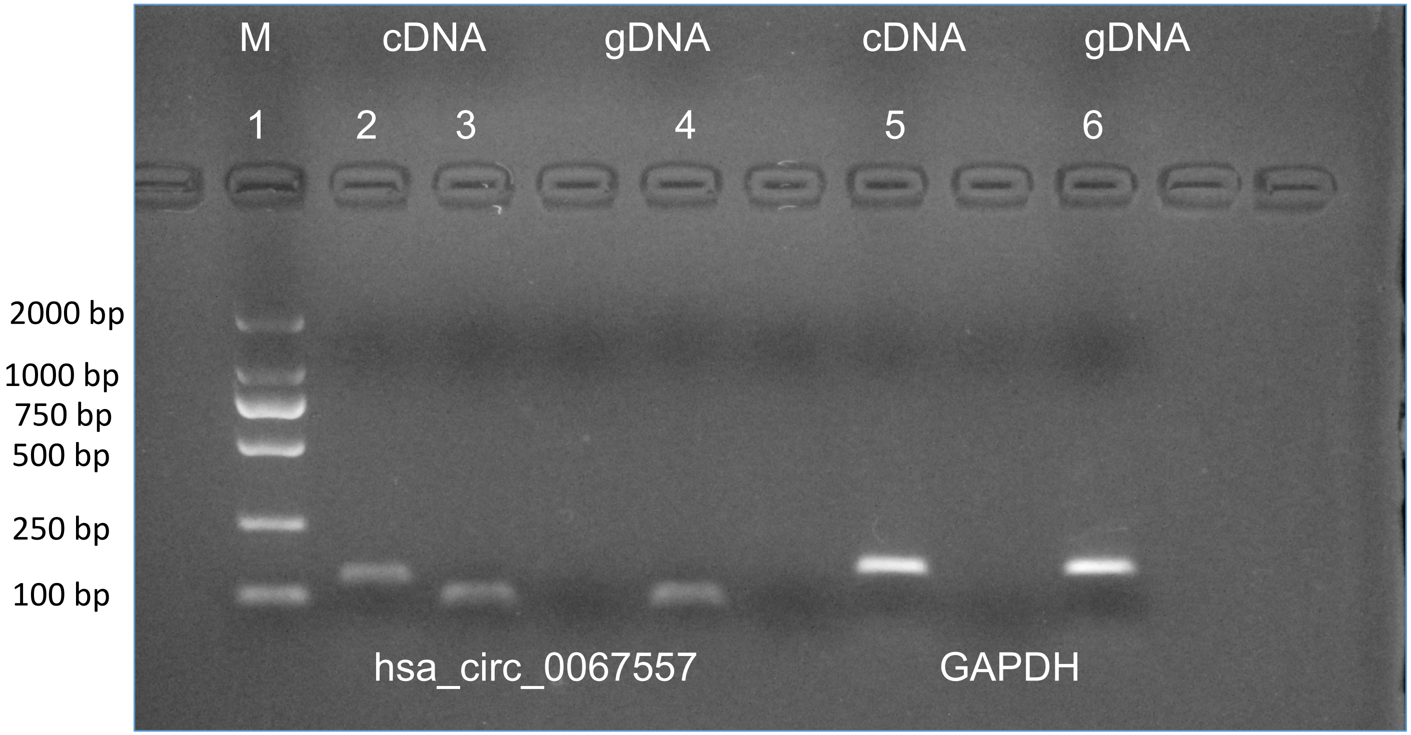

Original gels for Fig. 1J

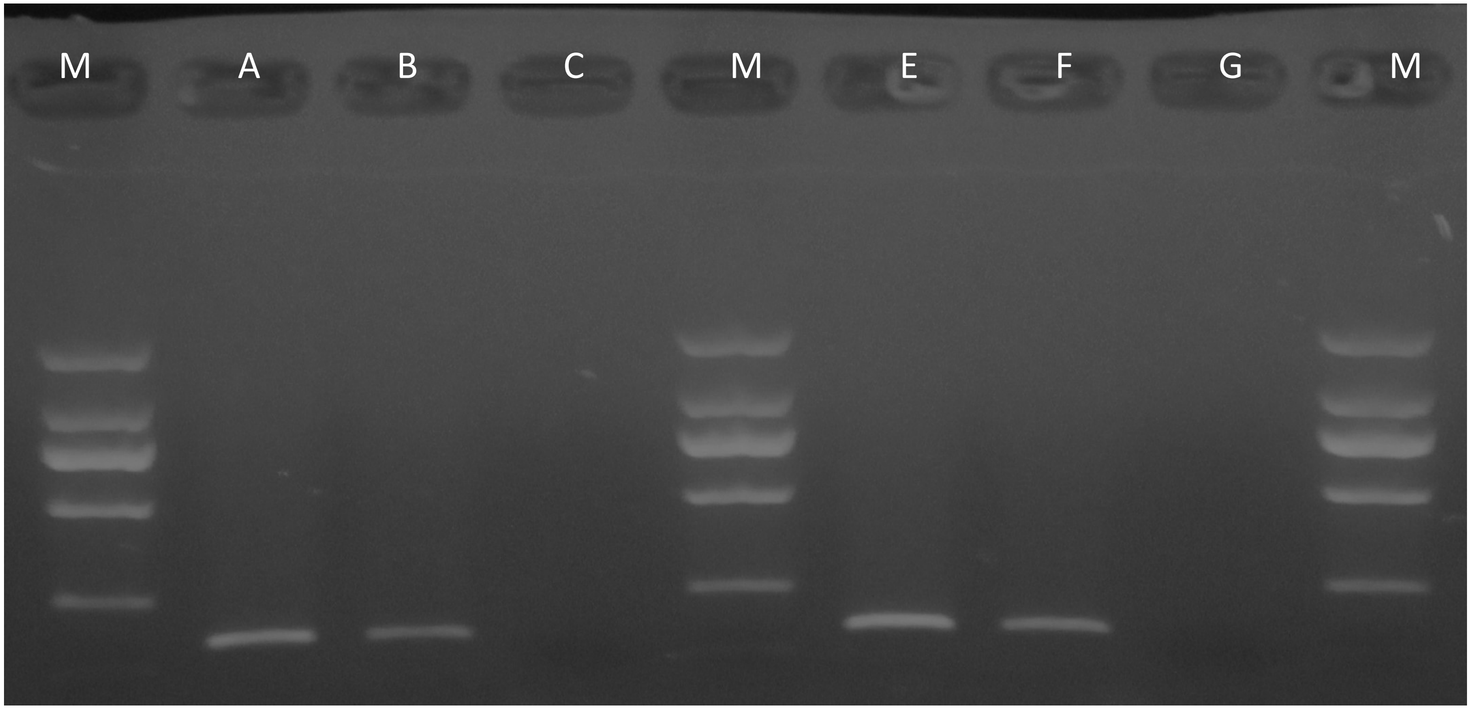

A:Input
B:IP-Lin28A
C:IgG

E:Input
F:IP-Lin28B
G:IgG

Original gels for Fig. 5A
