# Supplementary material for: Exosomes derived from cancer-associated fibroblasts promote tumorigenesis, metastasis and chemoresistance of colorectal cancer by upregulating circ_0067557 to target Lin28
Source: BMC Cancer. 2024 Jan 12;24:64. doi: 10.1186/s12885-023-11791-5 (PMC10785442; doi:10.1186/s12885-023-11791-5)
Supplement: Supplementary file 4 — Supplementary Material 4: The permission of KEGG [file 12885_2023_11791_MOESM4_ESM.pdf]

Ref: 231946

Permission is granted to BMC Cancer to publish under the CC BY 4.0 open access license the following KEGG pathway map image in the article "Exosomes derived from cancer-associated fibroblasts promote tumorigenesis, metastasis and chemoresistance of colorectal cancer by upregulating circ_0067557 to target Lin28" written by Zhiwei Yu and colleagues:

- Metabolic pathways (map01100)

subject to the condition that the original source is acknowledged by citing at least one KEGG paper.

Permission granted: Naomi Takahashi

Naomi Takahashi, Kanehisa Laboratories

Date: 19 October 2023

Copyright holder: Kanehisa Laboratories
